# Novel Phenoxyalkanoic Acid Derivatives as Free Fatty Acid Receptor 4 Agonists for Treating Type 2 Diabetes Mellitus

**DOI:** 10.3390/ijms252111476

**Published:** 2024-10-25

**Authors:** Xu Li, Xinmeng Zhang, Xueyuan Xie, Taimin Dong, Chengxu Lv, Ranran Guan, Wenyue Zhang, Guoxia Ji, Fanghui Chen, Shiben Wang, Xuekun Wang

**Affiliations:** 1 National Key Laboratory of Macromolecular Drug Development and Manufacturing, School of Pharmaceutical Sciences and Food Engineering, Liaocheng University, 1 Hunan Street, Liaocheng 252059, China; lx221024@126.com (X.L.); 18241016451@163.com (X.Z.); 18863356238@163.com (X.X.); 17616455575@163.com (T.D.); 17861811130@163.com (C.L.); grr19819342198@163.com (R.G.); z19806100327@163.com (W.Z.); chenfanghui@lcu.edu.cn (F.C.); 2 School of Chemistry and Chemical Engineering, Liaocheng University, 1 Hunan Street, Liaocheng 252059, China; jiguoxia@lcu.edu.cn

**Keywords:** free fatty acid receptor 4 agonists, design, synthesis, evaluation, type 2 diabetes mellitus

## Abstract

Diabetes mellitus (DM) is a common metabolic disease that poses a severe threat to human health. Despite a range of therapeutic approaches, there remains a lack of effective and safe therapies with the existing drugs. Therefore, there is an urgent need to develop novel, effective, and safe therapeutic strategies for DM. Free fatty acid receptor 4 (FFAR4), also known as GPR120, is a member of the G protein-coupled receptor family, which has received considerable attention as an attractive new therapeutic target for treating DM. In the present study, based on the structure of TUG-891, which has excellent activity and selectivity, a series of novel FFAR4 agonists was designed by replacing the phenylpropanoic acid β position carbon atom with an oxygen atom, while replacing the linking oxymethylene with an amide-linking group. The target compounds were evaluated for FFAR4 agonistic activity, and the preferred compounds were evaluated for selectivity, oral glucose tolerance in normal ICR mice, antidiabetic activity in diet-induced obese (DIO) mice, pharmacokinetic properties in ICR mice and molecular modeling studies. The results showed that compound **10f** possessed excellent FFAR4 agonistic activity and selectivity, significantly improved glucose tolerance in normal ICR mice, lowered blood glucose and promoted insulin secretion in a dose-dependent manner in DIO mice, and showed favorable pharmacokinetic properties. These results indicate that compound **10f** may be a promising compound that deserves further structure–activity relationship and pharmacological studies for the development of antidiabetic drugs.

## 1. Introduction

Diabetes mellitus (DM), a metabolic disease characterized by hyperglycemia, has become a public health problem that seriously affects human health and quality of life, and imposes a heavy economic burden on society [1]. According to statistics from the International Diabetes Federation, approximately 537 million adults worldwide had diabetes in 2021. It is estimated that these figures will increase to 642 million by 2040 and 783 million by 2045 [2]. Generally, DM can be divided into type 1 DM (T1DM), type 2 DM (T2DM), and gestational DM (GDM). T1DM is caused by an autoimmune condition that attacks the patient’s own insulin-producing β cells, and it accounts for approximately 10% of cases worldwide [3]. T2DM develops from gradual insulin insensitivity and a decrease in insulin production, and it accounts for approximately 90% of cases [4]. GDM is defined as a type of diabetes that is first diagnosed during pregnancy and shares a pathophysiology similar to that of T2DM. DM is accompanied by serious complications, such as diabetic nephropathy, diabetic retinopathy, diabetic neuropathy, cardiovascular complications, and liver fibrosis, which are causes of mortality in DM [5]. Common antidiabetic agents include insulin and its analogs, sulfonylureas, biguanides, thiazolidinediones, α-glucosidase inhibitors, meglitinides, sodium-glucose cotransporter 2 inhibitors, dipeptidyl peptidase IV inhibitors, and glucagon-like peptide-1 receptor agonists [6]. Despite the success of these agents in regulating blood glucose levels, most antidiabetic agents have adverse side effects including gastrointestinal disorders, anemia, renal failure, weight gain, and hypoglycemia [7]. Hence, there is a need to identify new antidiabetic agents that retain their therapeutic efficacy and are devoid of side effects.

The free fatty acid receptors (FFARs), such as FFAR1 (also known as GPR40), FFAR2 (also known as GPR43), FFAR3 (also known as GPR41), and FFAR4 (also known as GPR120), which belong to group A of the G protein-coupled receptor family, have become established as interesting potential therapeutic targets for treating metabolic diseases. FFARs are activated endogenously by free fatty acids of different chain lengths with varying levels of specificity. Among these groups of FFARs, FFAR1 and FFAR4 are activated by medium-chain fatty acids and long-chain fatty acids, whereas FFAR2 and FFAR3 are activated by short-chain fatty acids [8]. Human FFAR4, with a *10q23.33* chromosomal location, exists as two splice variants: a short isoform containing 361 residues and a long isoform containing 377 residues [9]. FFAR4 is expressed in the brain, pituitary gland, heart, liver, kidney, lung, stomach, muscle, small intestine, spleen, subcutaneous tissue, colon, pancreas, and other tissues, and it is most abundantly expressed in intestinal and adipose tissues [10,11]. FFAR4 participates in regulating multiple physiological functions in vivo, such as increasing intracellular Ca^2+^ concentration in response cells through the PLC (phospholipase C)/IP3 (inositol 1,4,5-trisphosphate) pathway, which promotes GLP-1 secretion by L cells [11,12,13,14], GIP secretion by K cells, and CCK secretion by I cells [15,16,17]. GLP-1, as an incretin hormone, can enhance glucose-dependent insulin release from pancreatic β-cells, delay gastric emptying, inhibit glucagon secretion, and promote β-cell proliferation [18,19]. Furthermore, when a ligand activates FFAR4, β-arrestin-2 can be recruited to the cell membrane, coupled with FFAR4, and then it can enter the cytoplasm through endocytosis, thereby inhibiting the binding of TAB1 and TAK1 and interfering with pro-inflammatory signaling [20]. Therefore, FFAR4 is an essential target in the treatment of DM, inflammation, and obesity.

**Figure 1:**
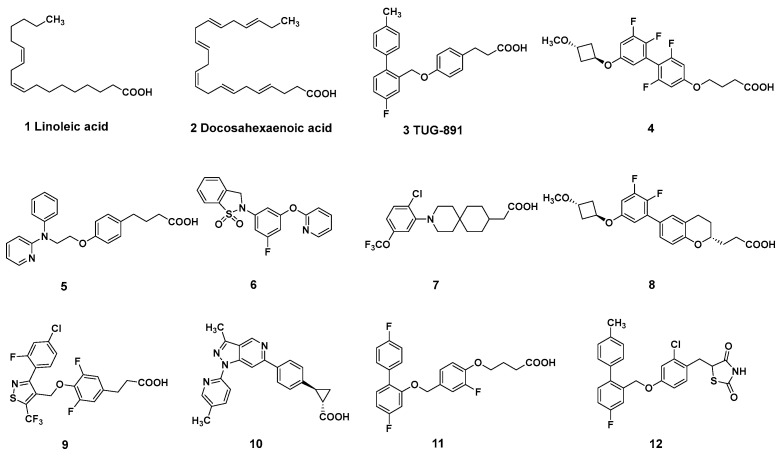
Representative free fatty acid receptor 4 agonists.

Although endogenous unsaturated long-chain fatty acids, such as linoleic acid (**1**) and docosahexaenoic acid (**2**) can activate FFAR4, they cannot be used as drugs because of their low activity and poor selectivity [21]. Much effort was made to develop novel FFAR4 agonists with high activity, selectivity, and safety, and many FFAR4 agonists were reported. However, there have been no clinical studies on these agents (Figure 1) [22,23,24,25,26,27,28,29,30,31,32,33,34,35,36,37]. Among these, TUG-891 was the first potent and selective FFAR4 agonist and was widely used to explore the physiological functions of FFAR4 [38,39,40,41]. Mao et al. determined the crystal structure of FFAR4 with saturated and unsaturated endogenous free fatty acids or TUG-891 using Cryo-EM, and the structures revealed that all fatty acid hormones and TUG-891 assumed an overall “L” configuration and were buried inside the seven transmembrane helix bundle of FFAR4 [42]. These crystal structures provide a foundation for the development of novel agonists with high activity and selectivity.

**Figure 2:**
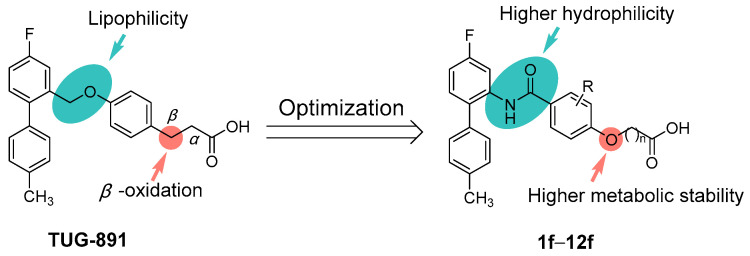
Compound design based on the bioisosteric replacement strategy.

TUG-891 showed good activity and selectivity; however, no clinical assessments were performed, suggesting that it may have some deficiencies to meet the requirements of clinical trials, such as poor pharmacokinetic properties due to high lipophilicity and β-oxidation of carboxylic acid [43,44,45]. In this study, novel FFAR4 agonists were designed based on the structure of TUG-891 to improve hydrophilicity by replacing the linking oxymethylene with an amide group and to improve metabolic stability by replacing the β-carbon atom with an oxygen atom. To elucidate the structure–activity relationship of the agonists, a series of novel phenoxyalkanoic acid derivatives were designed by introducing electron-donating or withdrawing substituents on the phenyl ring proximal to the carboxyl group and varying the length of carbon chains between the phenyl ring and the carboxyl group (Figure 2). The FFAR4 agonist activity was assessed in vitro, while hypoglycemic effects and pharmacokinetic properties were evaluated in vivo to identify compounds exhibiting enhanced agonistic activity, metabolic stability, and hydrophilicity.

## 2. Results

### 2.1. Chemistry

**Scheme 1:**
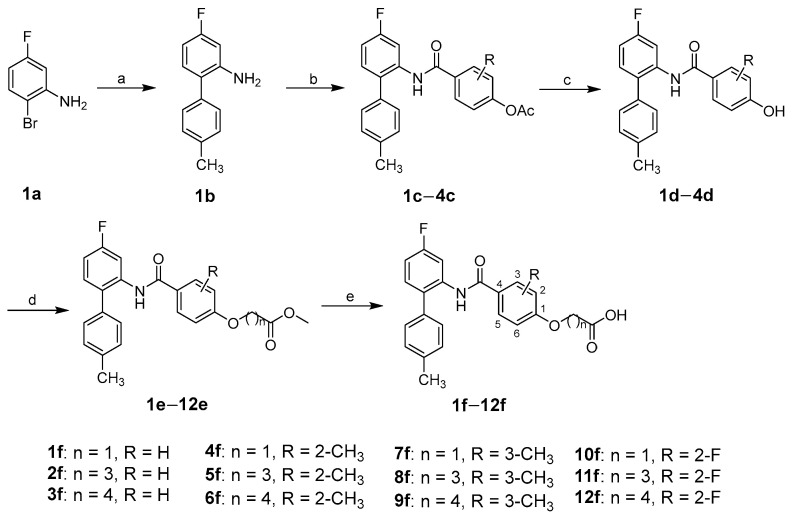
Reagents and conditions: (a) 4-tolylboronic acid, Pd(PPh_3_)_4_, Na_2_CO_3_, toluene, ethanol, H_2_O, 80 °C, overnight, 71%; (b) *p*-acetoxybenzoic acid or substituted *p*-acetoxybenzoic acid, EDCI, NEt_3_, CH_2_Cl_2_, rt, 12 h, 51–63%; (c) NaOH, H_2_O, CH_3_OH, THF, 4 h, 73–80%; (d) K_2_CO_3_, DMF, rt., 12 h; (e) LiOH, H_2_O, CH_3_OH, THF, 12 h, 46–65% (two steps).

The synthetic route for the target compounds **1f**–**12f** is shown in Scheme 1. Compounds 2-bromo-5-fluoroaniline **1a** and 4-tolylboronic acid reacted in a nitrogen atmosphere, catalyzed by palladium tetrakis (triphenylphosphine), to give intermediate **1b** through the Suzuki coupling reaction in 71% yield. The condensation of the amino group of intermediate **1b** with the carboxyl group of *p*-acetoxybenzoic acid or the substituted *p*-acetoxybenzoic acid occurred using 1-ethyl-3-(3-dimethylaminopropyl)carbodiimide hydrochloride as a condensation reagent and triethylamine as a base to obtain intermediates **1c**–**4c** in 51–63% yield. Intermediates **1c**–**4c** were hydrolyzed by NaOH to remove the acetyl protecting group to give intermediates **1d**–**4d** in 73–80% yield. The bromines in methyl bromoacetate, methyl bromobutyrate, or methyl bromovalerate were substituted with the phenolic hydroxyl groups of intermediates **1d**–**4d** via the Williamson ether synthesis to give intermediates **1e**–**12e** in the presence of potassium carbonate. The ester bonds of intermediates **1e**–**12e** were hydrolyzed with the weak base LiOH and then acidified to give target compounds **1f**–**12f** in 46–65% yield (over two steps).

### 2.2. Pharmacology

#### 2.2.1. FFAR4 Agonistic Activity and Selectivity

The designed and synthesized compounds were first evaluated for FFAR4 agonistic activity in vitro to determine the mechanisms of action using human FFAR4 transfected CHO cells, and TUG-891 was used as a positive control. The results showed that compound **1f**, obtained by replacing the oxymethylene with an amide bond and the phenylpropanoic acid β-carbon atom with an oxygen atom, reduced the FFAR4 agonistic activity nearly threefold. Compounds **2f** and **3f**, obtained by extending the length of the carbon chain between the carboxylic acid and oxygen, showed reduced FFAR4 activity compared to compound **1f** (**1f** > **2f** > **3f**). The agonistic activity of FFAR4 was largely retained in compound **4f** obtained after an introduction of the electron-donating methyl group at the 2-position of the benzene ring of compound **1f**. FFAR4 agonistic activity significantly increased after extending the length of the linking moiety between the carboxyl and oxygen groups of compound **4f**; however, FFAR4 agonistic activity decreased with further extension of the length (**5f** > **4f** > **6f**). Compound **7f**, which was obtained by introducing a methyl group at the 3-position of the benzene ring of compound **1f**, reduced FFAR4 agonistic activity. The FFAR4 agonistic activity was further reduced after prolonging the length of carbon chains (**7f** > **8f** > **9f**). The FFAR4 agonistic activity of compound **10f**, obtained by introducing an electron-withdrawing fluorine atom at the 2-position of the benzene ring of compound **1f**, was significantly higher than that of compound **1f**. FFAR4 agonistic activity was reduced after extending the length of the linking group (**10f** > **11f** > **12f**) (Table 1). Compound **10f** with good hFFAR4 agonistic activity was examined for human GPR40 (hGPR40) agonistic activity using human FFAR1 transfected CHO cells. The results revealed that the EC_50_ of compound **10f** and TUG-891 to hFFAR1 were 32.9 µM and 28.1 µM, respectively, indicating that compound **10f** had excellent selectivity.

The hydrophobicity of all compounds was predicted through cLogP values and topological polar surface area (TPSA) using the SwisADME online tool. The cLogP predictions indicated that compounds **6f**, **9f**, and **12f** exhibited higher cLogP values compared to the positive control, TUG-891, while the remaining compounds demonstrated lower cLogP values than TUG-891. Notably, compound **10f**, which displayed superior in vitro FFAR4 agonistic activity, possessed a cLogP value of 4.35. Furthermore, the TPSA of TUG-891 was measured at 46.53, whereas the synthesized compounds presented a higher TPSA of 75.63 (Table 1).

#### 2.2.2. Molecular Docking

**Figure 3:**
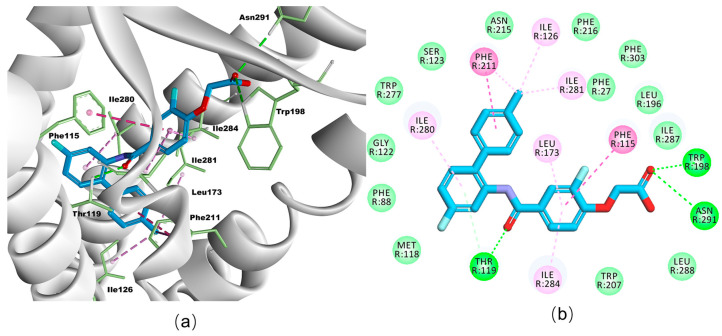
Molecular docking results of compound **10f** with free fatty acid receptor 4 (PDB code 8G59). (**a**) A 3D pose of compound **10f** with free fatty acid receptor 4 (protein is shown in gray, compound **10f** in blue and amino acid residues in green), and (**b**) a 2D plot of compound **10f** with free fatty acid receptor 4 (green circles and lines represent conventional hydrogen bonds, pink circles and lines represent π–π interactions, and light pink circles and lines represent π–alkyl interactions).

Compound **10f** exhibited the best FFAR4 agonistic activity among the synthesized compounds. To investigate the mode of interaction of the synthesized compounds with FFAR4, molecular docking was performed using BIOVIA Discovery Studio software v21.1.0.20298 to explain the potential intermolecular interactions between compound **10f** and FFAR4. Molecular docking results confirmed that compound **10f** interacted well with FFAR4, and the combinatory effect was relatively steady. The carboxyl group of compound **10f** formed hydrogen bonds with TRP198 and ASN291, which played a vital role in stabilizing its binding mode. In addition, the amide group of compound **10f** was able to form a hydrogen bond with THR119, which is advantageous for stabilizing the structures of the complexes. In addition, the aromatic ring moieties of **10f** exhibited several π-π and π–alkyl interactions with the pocket amino acids of FFAR4, including PHE115, PHE211, ILE280, and ILE284 (Figure 3a,b).

#### 2.2.3. Oral Glucose Tolerance Test (OGTT) of Compounds **5f**, **10f**, and **11f** in Normal ICR Mice

**Figure 4:**
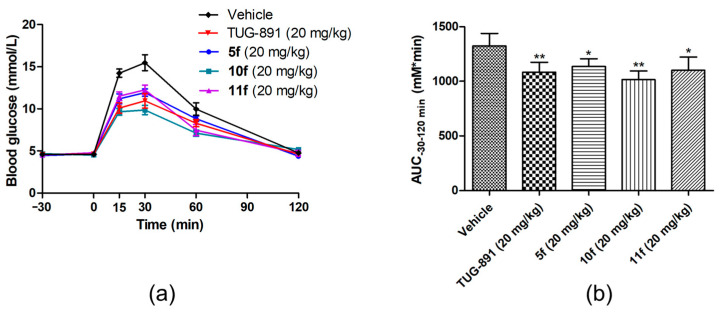
Effects of compounds **5f**, **10f**, **11f**, and TUG-891 on blood glucose levels during the oral glucose tolerance test in fasting ICR mice. (**a**) Time-dependent changes in blood glucose levels after oral administration of compounds, followed by the oral glucose load (2 g/kg). (**b**) AUC_−30–120 min_ of blood glucose levels. Values are expressed as the mean ± SD (*n* = 8). * *p* ≤ 0.05 compared with vehicle-treated group by Student’s *t* test; ** *p* ≤ 0.01 compared with vehicle-treated group by Student’s *t* test.

Compounds (20 mg/kg) or the vehicle was administered by gavage 30 min before glucose administration (2 g/kg). Blood glucose levels were measured using a glucometer before compound administration and glucose gavage and at 15, 30, 60, and 120 min after glucose gavage. The results showed that the blood glucose levels of ICR mice were within the range of 4–5 mmol/L after 12 h of fasting, and there was no significant change in the blood glucose levels after 30 min of compound administration. Blood glucose levels began to increase after the intragastric administration of glucose and peaked at 30 min in each group, after which the blood glucose began to decline and reached normal levels at 120 min. The area under the blood glucose–time curve (AUC) between 0 and 120 min was calculated. Compounds **5f**, **10f**, **11f,** and TUG-891 reduced the AUC by 16.7%, 30.8%, 20.0%, and 22.4%, respectively, compared with the vehicle-treated group (Figure 4a,b).

#### 2.2.4. Anti-Hyperglycemic Effects of Compound **10f** in Diet-Induced Obese (DIO) Mice

**Figure 5:**
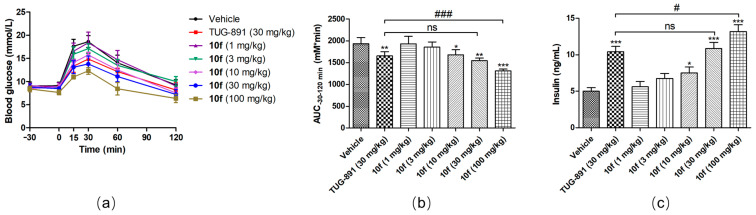
Effects of compound **10f** and TUG-891 on blood glucose levels during the oral glucose tolerance test in fasting DIO mice. (**a**) Time-dependent changes in blood glucose levels after oral administration of compound **10f**, followed by the oral glucose load (2 g/kg). (**b**) AUC_−30–120 min_ of blood glucose levels. (**c**) Insulin levels at 30 min after the glucose load. Values are expressed as the mean ± SD (*n* = 6). * *p* ≤ 0.05 compared with vehicle-treated group by Student’s *t* test; ** *p* ≤ 0.01 compared with vehicle-treated group by Student’s *t* test; *** *p* ≤ 0.001 compared with vehicle-treated group by Student’s *t* test; ^#^ *p* ≤ 0.05 compared with TUG-891-treated group by Student’s *t* test; ^###^ *p* ≤ 0.001 compared with TUG-891-treated group by Student’s *t* test; ns means not significant.

The blood glucose of DIO mice was in the range of 8–9 mmol/L after 12 h of fasting, which was significantly higher than the fasting blood glucose of normal mice. The compound **10f** was administered at 1, 3, 10, 30, and 100 mg/kg orally 30 min prior to a glucose challenge of 2 g/kg at a 10 mL/kg dose volume; the control groups were administered a single dose of either vehicle or TUG-891 (30 mg/kg) orally. Glucose levels were measured 30 min before and 120 min after the glucose challenge. Blood glucose levels rapidly increased after glucose administration and reached a maximum after 30 min. Subsequently, blood glucose levels began to decrease and reached normal levels in the **10f**-treated (30 and 100 mg/kg) and TUG-891-treated groups at 120 min, while levels remained high in the vehicle-treated group. The AUC between −30 and 120 min was calculated, and the results showed that compound **10f** reduced blood glucose levels in DIO mice in a dose-dependent manner. Treatment with 30 and 100 mg/kg of compound **10f** reduced the AUC by 20.1% and 32.4%, respectively, compared with the vehicle-treated group, and no hypoglycemia was observed during the experiment (Figure 5a,b). During the experimental period, plasma insulin levels were measured 30 min after glucose administration using an insulin ELISA Kit. The results showed that the insulin levels of the **10f** group with an administration of doses greater than 10 mg/kg were significantly higher than that of the control group (Figure 5c).

#### 2.2.5. Pharmacokinetic Evaluation of Compound **10f** in ICR Mice

The pharmacokinetic parameters of the maximum drug concentration in plasma (*C*_max_), the time to reach maximum drug concentration in plasma (*T*_max_), and the AUC from 0 to 24 h were evaluated in ICR mice. Table 2 shows *C*_max_, *T*_max,_ and the AUC values after the oral administration of 10 mg/kg of **10f** or TUG-891. The results showed that compound **10f** and TUG-891 reached *C*_max_ 30 min after oral administration, and the *C*_max_ of compound **10f** was 3.14 µg/mL, which is 2.5 times higher than that of TUG-891. The AUC was calculated using the trapezoidal method. The mean AUC value for **10f** was 9.83 µg·h/mL, which was significantly higher (approximately four fold) than that of TUG-891. In addition, the half-life of compound **10f** was prolonged compared with that of TUG-891.

## 3. Discussion

### 3.1. Chemistry

In this study, the target compounds **1f**–**12f** were synthesized in five steps, including Suzuki coupling, condensation reactions, deprotection, Williamson ether synthesis and hydrolysis in relatively good yields. Suzuki coupling is a powerful synthetic method for biphenyl derivatives, which can realize the cross-coupling of aryl or alkenyl boronic acids or boronic esters with chloro-, bromo-, and iodo-substituted aromatics or olefins catalyzed by zero-valent palladium complexes [46]. Spectroscopic methods (^1^H NMR and ^13^C NMR) were applied to confirm the chemical structure of target compounds. In the ^1^H NMR spectra, researchers should look for the singlet signal for the proton of the NH group at δ 9–10 ppm and another singlet signal for the proton of the COOH group in the range of δ 12–14 ppm. In the ^13^C NMR spectra, the amides show the signal of the carbon atom of the carbonyl group at around δ 165–170 ppm, and carboxylic acid shows the signal of the carbon atom of the carbonyl group in the range of δ 170–175 ppm. The chemical shift values for the analyzed fragments of the target compounds were in comparable ranges, which proved the proper synthesis and chemical structure of the obtained substances. Additional signals for other aliphatic and aromatic groups of the analyzed compounds were found at optimal and expected values of the chemical shift (δ).

### 3.2. Pharmacology Evaluatin

#### 3.2.1. In Vitro Evaluation

In vivo, the endogenous ligands of FFAR4 are free fatty acids, whose carboxyl groups are capable of forming hydrogen bonds with FFAR4, a critical aspect of their interaction. Consequently, the majority of FFAR4 agonists that were developed incorporated carboxylic acids or bioisosteres. Accordingly, the compounds designed in the current study preserved the carboxylic acid moiety. The structure–activity relationship studies demonstrated that the designed compounds exhibited retained FFAR4 agonistic activity. Compounds incorporating an electron-withdrawing group at the 2-position of the benzene ring displayed greater activity compared to those with either unsubstituted or electron-donating group substitutions. Furthermore, in compounds with either unsubstituted benzene rings, 3-methyl substitutions, or 2-fluorine substitutions, an increase in the carbon chain length between the carboxyl group and the oxygen atom resulted in a decrease in activity. Within the group of 2-position methyl-substituted compounds, the derivative of phenoxybutyric acid demonstrated superior activity. Modifying the carbon chain length between the oxygen atom and the carboxyl group, either by elongation or reduction, resulted in diminished activity. Among the synthesized compounds, compound **10f** exhibited the most potent FFAR4 agonistic activity. FFAR1 and FFAR4 were activated by medium-chain and long-chain fatty acids, and the selectivity of compound **10f** for FFAR4 over FFAR1 was investigated [47]. The results indicated that compound **10f** demonstrated approximately 400-fold greater potency in stimulating FFAR4 than FFAR1.

The hydrophobicity of all compounds was predicted using the SwisADME online tool. This tool provides the average cLogP value from the iLogP, xLogP, wLogP, mLogP, and silicos-it values [48]. A suitable cLogP is beneficial for compound druggability [43]. In addition, a calculation of the TPSA showed that all the synthesized compounds had higher polar surface area values than TUG-891, suggesting that the addition of an amide group increased the hydrophilicity of the structures. Among the compounds, compound **10f** showed higher hydrophilicity with a lower cLogP value and a higher TPSA value than TUG-891, suggesting that compound **10f** has better druggability properties.

#### 3.2.2. Molecular Docking Simulation

Molecular docking was employed to explain the potential intermolecular interactions between compound **10f** and FFAR4. The binding position of TUG-891 in the ligand pocket of FFAR4 was reported (PDB code 8G59), and the carboxyl group of TUG-891 can form conventional hydrogen-bonding interactions with TRP198 and ASN291, whereas the three aromatic rings of TUG-891 can interact with MET118, ILE280, ILE284, and ILE287 via amide Pi-stacked or Pi-alkyl interactions (Figure S1a,b) [40,42]. The docking results showed that compound **10f** interacted with FFAR4 in a manner similar to that of TUG-891, which indicated that the mechanism of action compound **10f** was consistent with that of TUG-891 through the activation of FFAR4.

#### 3.2.3. Evaluation in Normal ICR Mice

Among the designed compounds, **5f**, **10f**, and **11f** exhibited superior FFAR4 agonistic activity, albeit not as potent as TUG-891. However, these compounds demonstrated improved hydrophilicity, as indicated by their lower clogP values. Then, compounds **5f**, **10f**, and **11f** were evaluated in an oral glucose tolerance assay using normal ICR mice. The dosage (20 mg/kg) administered to mice was established through preliminary experiments (Figure S2). Compounds **5f**, **10f**, and **11f** were effective in improving oral glucose tolerance in normal ICR mice at a dose of 20 mg/kg, with compound **10f** showing the best effect, similar to that of TUG-891.

#### 3.2.4. Evaluation in DIO Mice

Compound **10f**, which significantly improved glucose tolerance in normal ICR mice, was selected to evaluate its antidiabetic effects in DIO mice, and TUG-891 was selected as a positive control. At a dosage of 30 mg/kg, TUG-891 significantly decreased blood glucose levels in DIO mice, evidenced by a 16.5% reduction in the AUC compared to the vehicle-treated group. Compound **10f** did not exhibit significant hypoglycemic effects at lower doses (1 mg/kg and 3 mg/kg). However, at doses exceeding 10 mg/kg, compound **10f** demonstrated significant hypoglycemic activity, as indicated by a notable reduction in the AUC relative to the vehicle-treated group. Additionally, a significant increase in insulin secretion was observed at these higher doses. Compound **10f** demonstrated an absence of hypoglycemic side effects at dosages up to 100 mg/kg, indicating that its mechanism of action in reducing blood glucose levels is glucose-stimulated insulin secretion, consistent with the action of GLP-1 [49]. These findings suggest that compound **10f** possesses a favorable safety profile.

#### 3.2.5. Pharmacokinetic Evaluation

The aim of this study was to improve the metabolic stability of TUG-891, and the pharmacokinetic properties of compound **10f** were evaluated in ICR mice. Previous research demonstrated that phenoxyalkanoic acid derivatives effectively enhance the stability of phenylpropanoic acid derivatives [50]. In this study, we investigated the pharmacokinetic properties of compound **10f**, which exhibited superior activity in both in vitro and in vivo assessments. Relative to the positive control TUG-891, compound **10f** demonstrated a significant increase in *C*_max_ and the AUC, as well as a notable extension of the *T*_1/2_. These findings suggest that compound **10f** possesses more advantageous pharmacokinetic properties.

## 4. Materials and Methods

### 4.1. Synthesis

All materials used were of the commercially available analytical grade without purification unless otherwise indicated. Silica gel (200–300 mesh, Qingdao Marine Chemical Company, Qingdao, China) was used for purification by column chromatography. The structures of the compounds **1f**–**12f** were unambiguously assessed by ^1^H NMR, ^13^C NMR, and high resolution mass spectrometry (HRMS). ^1^H NMR and ^13^C NMR spectra were recorded using tetramethylsilane (TMS) as the internal standard in dimethyl sulfoxide (DMSO)-*d*_6_ (Energy Chemical, Shanghai, China) with a Bruker AVANCE NEO 500 instrument (Bruker Biospin GmbH, Ettlingen, Germany) at 500 MHz and 125 MHz, respectively. The chemical shifts were reported in ppm relative to TMS as the internal standard, and coupling constants were measured in Hz. HRMS was conducted using a UPLC G2-XS QTOF spectrometer (Waters Corporation, Milford, MA, USA) with the electrospray ionization Fourier transform ion cyclotron resonance technique. The target compounds were ≥95% pure by HPLC analysis (Agilent Technologies, Santa Clara, CA, USA). The NMR, HRMS, and HPLC spectra of compounds **1f**–**12f** are presented in Supporting Materials (Figures S3–S50).

#### 4.1.1. Synthetic Procedure for Intermediate 4-Fluoro-4′-Methyl-[1,1′-Biphenyl]-2-Amine (**1b**)

2-Bromo-5-fluoroaniline (1.0 equiv) and *p*-tolueneboronic acid (1.0 equiv) were dissolved in 20 mL of 1N sodium carbonate solution, 10 mL of ethanol, and 20 mL of toluene. Pd(PPh_3_)_4_ (0.05 equiv) was added as a catalyst under nitrogen protection. The reaction mixture was refluxed under nitrogen at 80 °C for 12 h and cooled to room temperature after completion, monitored by thin layer chromatography (TLC). After dilution with 50 mL of water and 50 mL of ethyl acetate, the insoluble material was removed by filtration over a bed of celite. The organic phase was washed with water (2 × 50 mL) and saturated sodium chloride (2 × 50 mL). Then, the organic phase was dried with anhydrous Na_2_SO_4_, filtered, and concentrated under reduced pressure. The residue was purified by column chromatography on silica gel to give the desired intermediate 0.75 g.

^1^H NMR (500 MHz, DMSO-*d*_6_) δ 7.29–7.21 (m, 4H), 6.95 (dd, *J* = 8.4, 6.9 Hz, 1H), 6.54 (dd, *J* = 11.7, 2.7 Hz, 1H), 6.39 (td, *J* = 8.5, 2.7 Hz, 1H), 5.05 (s, 2H), 2.34 (s, 3H).

#### 4.1.2. General Synthetic Procedure for Intermediates **1c**–**4c**

To a solution of 4-acetoxybenzoic acid (1.0 equiv) or substituted 4-acetoxybenzoic acid (1.0 equiv) in 30 mL of dry dichloromethane, triethylamine (2.0 equiv) and 1-ethyl-3-(3-dimethylaminopropyl) carbodiimide (EDCI, 1.5 equiv) were added at room temperature. After stirring for 30 min, 5 mL of dry dichloromethane dissolved, and **1b** (1.0 equiv) was added slowly with stirring. The mixture was stirred at room temperature and quenched with water (50 mL) after TLC indicated the completion of the reaction. The mixture was extracted with dichloromethane (100 mL) and washed with water (2 × 50 mL) and saturated sodium chloride (2 × 50 mL). The organic phase was dried with anhydrous Na_2_SO_4_, filtered, and concentrated under reduced pressure. The residue was purified by column chromatography on silica gel to give the desired corresponding intermediates **1c**–**4c**.

#### 4.1.3. General Synthetic Procedure for Intermediates **1d**–**4d**

To a solution of the corresponding intermediates **1c**–**4c** (1.0 equiv) in 20 mL THF and 20 mL methanol, 2 mol/L sodium hydroxide (4.0 equiv) was added at room temperature. The mixture was stirred at room temperature for 4 h and acidified with dilute hydrochloric acid to pH 4–5 after TLC indicated the completion of the reaction. The mixture was extracted with ethyl acetate (2 × 50 mL), and the combined organic phases were washed with water (2 × 50 mL) and saturated sodium chloride (2 × 50 mL). The organic phase was dried with anhydrous Na_2_SO_4_, filtered, and concentrated under reduced pressure. The residue was purified by column chromatography on silica gel to give the desired corresponding phenol intermediates **1d**–**4d**.

N-(4-fluoro-4′-methyl-[1,1′-biphenyl]-2-yl)-4-hydroxybenzamide (**1d**): ^1^H NMR (500 MHz, Chloroform-*d*) δ 8.40 (dd, *J* = 11.4, 2.7 Hz, 1H), 8.01 (s, 1H), 7.51 (d, *J* = 8.6 Hz, 2H), 7.33 (d, *J* = 8.0 Hz, 2H), 7.29 (d, *J* = 8.1 Hz, 1H), 7.21 (dd, *J* = 8.4, 6.3 Hz, 1H), 6.88 (td, *J* = 8.2, 2.7 Hz, 1H), 6.82 (d, *J* = 8.7 Hz, 2H), 2.44 (s, 3H).

N-(4-fluoro-4′-methyl-[1,1′-biphenyl]-2-yl)-4-hydroxy-3-methylbenzamide (**2d**): ^1^H NMR (500 MHz, DMSO-*d*_6_) δ 9.98 (s, 1H), 9.35 (s, 1H), 7.52 (d, *J* = 2.3 Hz, 1H), 7.52–7.44 (m, 2H), 7.36 (dd, *J* = 8.6, 6.5 Hz, 1H), 7.31 (d, *J* = 8.1 Hz, 2H), 7.22 (d, *J* = 7.9 Hz, 2H), 7.15 (td, *J* = 8.4, 2.7 Hz, 1H), 6.79 (d, *J* = 8.4 Hz, 1H), 2.31 (s, 3H), 2.12 (s, 3H).

N-(4-fluoro-4′-methyl-[1,1′-biphenyl]-2-yl)-4-hydroxy-2-methylbenzamide (**3d**): ^1^H NMR (500 MHz, DMSO-*d*_6_) δ 9.70 (s, 1H), 9.32 (s, 1H), 7.48 (dd, *J* = 10.5, 2.7 Hz, 1H), 7.33 (dd, *J* = 8.6, 6.5 Hz, 1H), 7.29 (d, *J* = 8.1 Hz, 2H), 7.23 (s, 1H), 7.21 (d, *J* = 8.0 Hz, 2H), 7.15 (td, *J* = 8.4, 2.8 Hz, 1H), 6.60 (d, *J* = 2.4 Hz, 1H), 6.57 (dd, *J* = 8.3, 2.5 Hz, 1H), 2.32 (s, 3H), 2.21 (s, 3H).

3-fluoro-N-(4-fluoro-4′-methyl-[1,1′-biphenyl]-2-yl)-4-hydroxybenzamide (**4d**): ^1^H NMR (500 MHz, DMSO-*d*_6_) δ 10.55 (s, 1H), 9.61 (s, 1H), 7.58 (dd, *J* = 12.2, 2.2 Hz, 1H), 7.50 (dd, *J* = 8.5, 2.1 Hz, 1H), 7.41 (dd, *J* = 10.4, 2.8 Hz, 1H), 7.38 (dd, *J* = 8.6, 6.5 Hz, 1H), 7.29 (d, *J* = 8.2 Hz, 2H), 7.20 (d, *J* = 7.8 Hz, 2H), 7.17 (dd, *J* = 8.5, 2.8 Hz, 1H), 6.99 (t, *J* = 8.6 Hz, 1H), 2.31 (s, 3H).

#### 4.1.4. General Synthetic Procedure for Intermediates **1e**–**12e**

To a solution of the phenol intermediates **1c**–**4c** (1.0 equiv) in 20 mL of dry DMF, the corresponding bromide (1.0 equiv), potassium carbonate (2.0 equiv) and a catalytic amount of potassium iodide (0.1 equiv) were added at room temperature. The mixture was stirred at room temperature for 12 h and concentrated under reduced pressure to remove DMF after TLC indicated the completion of the reaction. The residue was dissolved in 50 mL of ethyl acetate and washed with water (2 × 50 mL) and saturated sodium chloride (2 × 50 mL). The organic phase was dried with anhydrous Na_2_SO_4_, filtered, and concentrated under reduced pressure. The corresponding residues **1e**–**12e** were used in the next step without further purification.

#### 4.1.5. General Synthetic Procedure for Target Compounds **1f**–**12f**

To a solution of comprising intermediates **1e**–**12e** (1.0 equiv) in 10 mL THF and 10 mL methanol, 2 mol/L of weaker base lithium hydroxide (8.0 equiv) was added at room temperature. The mixture was stirred at room temperature for 12 h and acidified with dilute hydrochloric acid to pH 4–5 after TLC indicated the completion of the reaction. The mixture was extracted with ethyl acetate (2 × 30 mL), and the combined organic phases were washed with water (2 × 50 mL) and saturated sodium chloride (2 × 30 mL). The organic phase was dried with anhydrous Na_2_SO_4_, filtered, and concentrated under reduced pressure. The residue was purified by column chromatography on silica gel to give desired target compounds **1f**–**12f**.

2-(4-((4-fluoro-4′-methyl-[1,1′-biphenyl]-2-yl)carbamoyl)phenoxy)acetic acid (**1f**): Colorless solid 0.11 g, yield 58% of two steps; ^1^H NMR (500 MHz, DMSO-*d*_6_) δ 9.59 (s, 1H), 7.73 (d, *J* = 8.8 Hz, 2H), 7.45 (dd, *J* = 10.4, 2.7 Hz, 1H), 7.38 (dd, *J* = 8.6, 6.5 Hz, 1H), 7.31 (d, *J* = 8.0 Hz, 2H), 7.20 (d, *J* = 7.9 Hz, 2H), 7.17 (dd, *J* = 8.4, 2.8 Hz, 1H), 6.94 (d, *J* = 8.8 Hz, 2H), 4.62 (s, 2H), 2.30 (s, 3H); ^13^C NMR (125 MHz, DMSO-*d*_6_) δ 165.48, 162.43, 161.26, 160.50, 137.11, 137.00, 135.70, 134.02, 132.11, 129.69, 129.48, 129.05, 126.89, 114.62, 114.51, 113.48, 65.82, 21.17; HRMS calcd for C_22_H_18_FNO_4_ [M-H]^−^ 378.1142, found 378.1140.

4-(4-((4-fluoro-4′-methyl-[1,1′-biphenyl]-2-yl)carbamoyl)phenoxy)butanoic acid (**2f**): Colorless solid 0.10 g, yield 49% of two steps; ^1^H NMR (500 MHz, DMSO-*d*_6_) δ 12.14 (s, 1H), 9.59 (s, 1H), 7.75 (d, *J* = 8.9 Hz, 2H), 7.46 (dd, *J* = 10.4, 2.8 Hz, 1H), 7.38 (dd, *J* = 8.6, 6.5 Hz, 1H), 7.31 (d, *J* = 8.1 Hz, 2H), 7.20 (d, *J* = 8.0 Hz, 2H), 7.17 (dd, *J* = 8.5, 2.8 Hz, 1H), 6.99 (d, *J* = 8.9 Hz, 2H), 4.04 (t, *J* = 6.5 Hz, 2H), 2.38 (t, *J* = 7.3 Hz, 2H), 2.00–1.90 (m, 2H); ^13^C NMR (125 MHz, DMSO-*d*_6_) δ 174.50, 165.47, 161.68, 160.50, 136.98, 135.70, 132.10, 129.86, 129.07, 126.70, 114.56, 114.38, 113.50, 67.36, 30.51, 24.59, 21.17; HRMS calcd for C_24_H_22_FNO_4_ [M-H]^−^ 406.1455, found 406.1453.

5-(4-((4-fluoro-4′-methyl-[1,1′-biphenyl]-2-yl)carbamoyl)phenoxy)pentanoic acid (**3f**): Colorless solid 0.11 g, yield 52% of two steps; ^1^H NMR (500 MHz, DMSO-*d*_6_) δ 12.02 (s, 1H), 9.58 (s, 1H), 7.75 (d, *J* = 8.8 Hz, 2H), 7.46 (dd, *J* = 10.4, 2.8 Hz, 1H), 7.38 (dd, *J* = 8.6, 6.5 Hz, 1H), 7.31 (d, *J* = 8.1 Hz, 2H), 7.20 (d, *J* = 8.0 Hz, 2H), 7.17 (dd, *J* = 8.5, 2.8 Hz, 1H), 6.99 (d, *J* = 8.8 Hz, 2H), 4.03 (t, *J* = 6.3 Hz, 2H), 2.30 (s, 3H), 2.28 (t, *J* = 7.3 Hz, 2H), 1.79–1.70 (m, 2H), 1.70–1.61 (m, 2H); ^13^C NMR (125 MHz, DMSO-*d*_6_) δ 174.79, 165.48, 137.13, 136.98, 135.70, 134.02, 132.03, 129.85, 129.47, 129.07, 126.61, 114.55, 113.48, 67.90, 33.71, 28.46, 21.62, 21.17; HRMS calcd for C_24_H_22_FNO_4_ [M-H]^−^ 4061455, found 406.1453.

2-(4-((4-fluoro-4′-methyl-[1,1′-biphenyl]-2-yl)carbamoyl)-2-methylphenoxy)acetic acid (**4f**): Colorless solid 0.09 g, yield 46% of two steps; ^1^H NMR (500 MHz, DMSO-*d*_6_) δ 13.03 (s, 1H), 9.53 (s, 1H), 7.62–7.56 (m, 2H), 7.46 (dd, *J* = 10.4, 2.8 Hz, 1H), 7.38 (dd, *J* = 8.6, 6.5 Hz, 1H), 7.31 (d, *J* = 8.0 Hz, 2H), 7.21 (d, *J* = 7.9 Hz, 2H), 7.17 (td, *J* = 8.4, 2.8 Hz, 1H), 6.89 (d, *J* = 8.5 Hz, 1H), 4.78 (s, 2H), 2.31 (s, 3H), 2.20 (s, 3H); ^13^C NMR (125 MHz, DMSO-*d*_6_) δ 170.40, 165.58, 162.45, 160.52, 159.04, 137.17, 137.03, 135.68, 133.86, 132.01, 130.54, 129.50, 129.09, 127.21, 126.84, 126.25, 114.26, 113.38, 111.23, 65.19, 21.17, 16.54; HRMS calcd for C_23_H_20_FNO_4_ [M-H]^−^ 392.1298, found 392.1299.

4-(4-((4-fluoro-4′-methyl-[1,1′-biphenyl]-2-yl)carbamoyl)-2-methylphenoxy)butanoic acid (**5f**): Colorless solid 0.11 g, yield 52% of two steps; ^1^H NMR (500 MHz, DMSO-*d*_6_) δ 12.13 (s, 1H), 9.49 (s, 1H), 7.61 (dd, *J* = 8.4, 2.4 Hz, 1H), 7.61–7.57 (m, 1H), 7.47 (dd, *J* = 10.4, 2.8 Hz, 1H), 7.37 (dd, *J* = 8.6, 6.5 Hz, 1H), 7.31 (d, *J* = 8.0 Hz, 2H), 7.21 (d, *J* = 7.9 Hz, 2H), 7.17 (td, *J* = 8.4, 2.8 Hz, 1H), 6.97 (d, *J* = 8.5 Hz, 1H), 4.05 (t, *J* = 6.3 Hz, 2H), 2.41 (t, *J* = 7.3 Hz, 2H), 2.31 (s, 3H), 2.16 (s, 3H), 2.02–1.94 (m, 2H); ^13^C NMR (125 MHz, DMSO-*d*_6_) δ 174.54, 165.61, 159.75, 137.21, 137.02, 135.68, 133.81, 132.06, 131.99, 130.35, 129.49, 129.11, 127.43, 126.21, 126.12, 114.22, 113.16, 111.02, 67.41, 30.67, 24.68, 21.17, 16.41; HRMS calcd for C_25_H_24_FNO_4_ [M-H]^−^ 420.1611, found 420.1608.

5-(4-((4-fluoro-4′-methyl-[1,1′-biphenyl]-2-yl)carbamoyl)-2-methylphenoxy)pentanoic acid (**6f**): Colorless solid 0.10 g, yield 46% of two steps; ^1^H NMR (500 MHz, DMSO-*d*_6_) δ 12.03 (s, 1H), 9.49 (s, 1H), 7.61 (dd, *J* = 8.6, 2.4 Hz, 1H), 7.59 (d, *J* = 2.2 Hz, 1H), 7.48 (dd, *J* = 10.4, 2.8 Hz, 1H), 7.37 (dd, *J* = 8.6, 6.5 Hz, 1H), 7.31 (d, *J* = 7.7 Hz, 2H), 7.21 (d, *J* = 7.8 Hz, 2H), 7.17 (td, *J* = 8.4, 2.8 Hz, 1H), 6.98 (d, *J* = 8.5 Hz, 1H), 4.04 (t, *J* = 6.2 Hz, 2H), 2.31 (s, 3H), 2.29 (t, *J* = 7.1 Hz, 2H), 1.80–1.72 (m, 2H), 1.72–1.63 (m, 2H); ^13^C NMR (125 MHz, DMSO-*d*_6_) δ 174.82, 160.52, 137.22, 137.02, 135.68, 133.79, 132.06, 130.33, 129.49, 127.43, 126.12, 126.04, 114.20, 113.31, 111.06, 67.94, 33.73, 28.55, 21.69, 21.17; HRMS calcd for C_26_H_26_FNO_4_ [M-H]^−^ 434.1768, found 434.1763.

2-(4-((4-fluoro-4′-methyl-[1,1′-biphenyl]-2-yl)carbamoyl)-3-methylphenoxy)acetic acid (**7f**): Colorless solid 0.11 g, yield 56% of two steps; ^1^H NMR (500 MHz, DMSO-*d*_6_) δ 12.93 (s, 1H), 9.53 (s, 1H), 7.46 (dd, *J* = 10.3, 2.8 Hz, 1H), 7.35 (dd, *J* = 8.6, 6.5 Hz, 1H), 7.30 (d, *J* = 8.0 Hz, 2H), 7.23 (d, *J* = 8.0 Hz, 2H), 7.17 (td, *J* = 8.4, 2.8 Hz, 1H), 6.78 (d, *J* = 2.6 Hz, 1H), 6.75 (dd, *J* = 8.4, 2.6 Hz, 1H), 4.70 (s, 2H), 2.32 (s, 2H), 2.24 (s, 2H); ^13^C NMR (125 MHz, DMSO-*d*_6_) δ 170.44, 168.03, 162.48, 160.54, 138.71, 137.12, 137.00, 135.85, 134.21, 132.10, 129.51, 129.37, 129.33, 129.20, 117.13, 114.29, 113.45, 111.43, 64.80, 21.18, 20.09; HRMS calcd for C_23_H_20_FNO_4_ [M-H]^−^ 392.1298, found 392.1300.

4-(4-((4-fluoro-4′-methyl-[1,1′-biphenyl]-2-yl)carbamoyl)-3-methylphenoxy)butanoic acid (**8f**): Colorless solid 0.12 g, yield 57% of two steps; ^1^H NMR (500 MHz, DMSO-*d*_6_) δ 12.10 (s, 1H), 9.46 (s, 1H), 7.47 (dd, *J* = 10.3, 2.7 Hz, 1H), 7.35 (dd, *J* = 8.6, 6.5 Hz, 1H), 7.30 (d, *J* = 8.2 Hz, 3H), 7.22 (d, *J* = 7.8 Hz, 2H), 7.16 (td, *J* = 8.5, 2.8 Hz, 1H), 6.79 (d, *J* = 2.5 Hz, 1H), 6.76 (dd, *J* = 8.4, 2.6 Hz, 1H), 3.99 (t, *J* = 6.4 Hz, 2H), 2.36 (t, *J* = 7.3 Hz, 2H), 2.32 (s, 3H), 2.25 (s, 3H), 1.92 (t, *J* = 6.9 Hz, 2H); ^13^C NMR (125 MHz, DMSO-*d*_6_) δ 174.50, 168.06, 160.55, 159.89, 138.85, 137.16, 137.01, 134.13, 132.08, 129.44, 129.38, 129.21, 128.93, 117.15, 114.19, 111.50, 67.11, 30.49, 24.61, 21.18, 20.15; HRMS calcd for C_25_H_24_FNO_4_ [M-H]^−^ 420.1611, found 420.1609.

5-(4-((4-fluoro-4′-methyl-[1,1′-biphenyl]-2-yl)carbamoyl)-3-methylphenoxy)pentanoic acid (**9f**): Colorless solid 0.12 g, yield 55% of two steps; ^1^H NMR (500 MHz, DMSO-*d*_6_) δ 12.00 (s, 1H), 9.46 (s, 1H), 7.47 (dd, *J* = 10.3, 2.8 Hz, 1H), 7.34 (dd, *J* = 8.6, 6.5 Hz, 1H), 7.30 (d, *J* = 7.9 Hz, 3H), 7.22 (d, *J* = 7.9 Hz, 2H), 7.16 (td, *J* = 8.4, 2.8 Hz, 1H), 6.78 (d, *J* = 2.5 Hz, 1H), 6.76 (dd, *J* = 8.4, 2.6 Hz, 1H), 3.98 (t, *J* = 6.3 Hz, 2H), 2.32 (s, 3H), 2.27 (t, *J* = 7.2 Hz, 2H), 2.25 (s, 3H), 1.75–1.68 (m, 2H), 1.67–1.59 (m, 2H); ^13^C NMR (126 MHz, DMSO-*d*_6_) δ 174.82, 168.07, 162.49, 160.02, 138.83, 137.01, 135.84, 134.11, 132.08, 129.43, 129.38, 129.21, 128.83, 117.15, 114.16, 111.48, 67.66, 33.74, 28.49, 21.63, 21.18, 20.16; HRMS calcd for C_26_H_26_FNO_4_ [M-H]^−^ 434.1768, found 434.1768.

2-(2-fluoro-4-((4-fluoro-4′-methyl-[1,1′-biphenyl]-2-yl)carbamoyl)phenoxy)acetic acid (**10f**): Colorless solid 0.13 g, yield 65% of two steps; ^1^H NMR (500 MHz, DMSO-*d*_6_) δ 9.79 (s, 1H), 7.65 (d, *J* = 12.6 Hz, 1H), 7.59 (d, *J* = 8.7 Hz, 1H), 7.47–7.35 (m, 2H), 7.30 (d, *J* = 7.6 Hz, 2H), 7.25–7.16 (m, 3H), 7.10 (t, *J* = 8.7 Hz, 1H), 4.72 (s, 2H), 2.30 (s, 3H); ^13^C NMR (125 MHz, DMSO-*d*_6_) δ 170.41, 164.53, 162.41, 160.48, 152.21, 150.27, 149.41, 136.99, 136.84, 135.70, 134.39, 132.16, 129.44, 129.01, 127.15, 124.84, 115.80, 114.91, 114.65, 113.83, 66.37, 21.15; HRMS calcd for C_22_H_18_F_2_NO_4_ [M+H]^+^ 398.1204, found 398.1203.

4-(2-fluoro-4-((4-fluoro-4′-methyl-[1,1′-biphenyl]-2-yl)carbamoyl)phenoxy)butanoic acid (**11f**): Colorless solid 0.11 g, yield 52% of two steps; ^1^H NMR (500 MHz, DMSO-*d*_6_) δ 12.18 (s, 1H), 9.74 (s, 1H), 7.63 (tt, *J* = 9.1, 2.3 Hz, 2H), 7.42 (dd, *J* = 10.2, 2.7 Hz, 1H), 7.39 (dd, *J* = 8.6, 6.5 Hz, 1H), 7.30 (d, *J* = 8.2 Hz, 2H), 7.26 (t, *J* = 8.7 Hz, 1H), 7.22–7.17 (m, 3H), 4.14 (t, *J* = 6.5 Hz, 2H), 2.40 (t, *J* = 7.3 Hz, 2H), 2.30 (s, 3H), 2.03–1.94 (m, 2H); ^13^C NMR (125 MHz, DMSO-*d*_6_) δ 174.40, 164.52, 162.42, 160.49, 152.36, 150.42, 149.73, 137.00, 136.81, 135.68, 134.33, 132.16, 129.44, 129.03, 127.09, 125.16, 115.75, 114.91, 114.66, 113.83, 68.46, 30.35, 24.49, 21.21; HRMS calcd for C_24_H_21_F_2_NO_4_Na [M+Na]^+^ 448.1336, found 448.1334.

5-(2-fluoro-4-((4-fluoro-4′-methyl-[1,1′-biphenyl]-2-yl)carbamoyl)phenoxy)pentanoic acid (**12f**): Colorless solid 0.13 g, yield 59% of two steps; ^1^H NMR (500 MHz, DMSO-*d*_6_) δ 12.05 (s, 1H), 9.73 (s, 1H), 7.64 (dd, *J* = 10.1, 2.3 Hz, 1H), 7.62 (t, *J* = 2.7 Hz, 1H), 7.43 (dd, *J* = 10.3, 2.8 Hz, 1H), 7.39 (dd, *J* = 8.6, 6.5 Hz, 1H), 7.30 (d, *J* = 8.2 Hz, 2H), 7.25 (t, *J* = 8.8 Hz, 1H), 7.22–7.17 (m, 3H), 4.12 (t, *J* = 6.4 Hz, 2H), 2.35–2.27 (m, 5H), 1.77 (dq, *J* = 8.4, 6.4 Hz, 2H), 1.66 (qd, *J* = 9.4, 8.5, 5.8 Hz, 2H); ^13^C NMR (125 MHz, DMSO-*d*_6_) δ 174.80, 164.54, 162.43, 160.49, 152.35, 150.41, 149.87, 136.99, 136.82, 135.68, 134.32, 132.16, 129.44, 129.04, 126.96, 125.15, 115.71, 114.89, 114.62, 113.82, 69.01, 33.67, 28.37, 21.51, 21.15; HRMS calcd for C_25_H_23_F_2_NO_4_Na [M+Na]^+^ 462.1493, found 462.1488.

### 4.2. Pharmacologic Studies

Male ICR mice and C57BL/6 mice four-weeks-old were purchased from Jinan Pengyue Experimental Animal Breeding Co., Ltd. (Jinan, China). The mice were housed in cages under a 12 h light/dark cycle from 7:00 to 19:00 at controlled temperatures (25–26 °C) and relative humidity (50 ± 10%) throughout the experimental period. All animals were allowed to eat and drink freely unless otherwise stated and were allowed to acclimatize for 1 week before the experiment. All animal experimental protocols were performed following applicable institutional and governmental regulations concerning the ethical use of animals.

#### 4.2.1. OGTT in Normal ICR Mice

Normal male ICR mice were weighed and randomly divided into five groups (eight mice per group) according to body weight after 12 h of fasting. The test compounds were dissolved in 0.5% methyl cellulose (MC) and vortexed before the study initiation. Mice in each group were gavaged with vehicle (0.5% MC aqueous solution, 10 mL/kg), TUG-891 (20 mg/kg, 10 mL/kg), or compounds **5f**, **10f**, **11f** (20 mg/kg; 10 mL/kg) 30 min before oral glucose loading (2 g/kg, 10 mL/kg). The exact dose volume was calculated separately for each animal. Blood samples were collected via the tail tip 30 min before the compound dose, at t = 0 (immediately before glucose loading), and 15, 30, 60, and 120 min after glucose loading. Blood glucose levels were measured using blood glucose test strips (Sannuo GA-3 type; Changsha, China). Glucose values were entered into an Excel sheet (version 2016, Microsoft, Redmond, WA, USA) and plotted using GraphPad Prism version 5.0 software (version 5.0, GraphPad, San Diego, CA, USA).

#### 4.2.2. Anti-Hyperglycemic Effects in DIO Mice

After 1 week of adaptation, male C57BL/6 mice were fed a high-fat diet (45% calories from fat, from Mediscience Ltd., Yangzhou, China) ad libitum for an additional 12 weeks to induce insulin resistance and were used as DIO mice. DIO mice were fasted overnight (12 h), weighed, and randomly divided into six groups (six mice per group) according to body weight and blood glucose level. Thereafter, the DIO mice were orally administered a single dose of vehicle (0.5% MC, 10 mL/kg), TUG-891 (30 mg/kg, 10 mL/kg), or **10f** (1, 3, 10, 30, 100 mg/kg; 10 mL/kg), 30 min before oral glucose loading (2 g/kg). Blood samples were collected via the tail tip or retro-orbital sinus puncture, and glucose levels were measured in accordance with the OGTT in normal ICR mice. Plasma insulin levels were measured using a mouse insulin RIA kit 30 min after oral glucose loading (Beijing North Institute of Biological Technology, Beijing, China).

#### 4.2.3. Pharmacokinetic Studies in ICR Mice

Pharmacokinetic studies of compounds **10f** was performed in male ICR mice eight-weeks-old, and TUG-891 was used as a positive control. Male ICR mice weighing 28–32 g were starved for 12 h and randomly divided into three groups (four mice per group). Compound **10f** and TUG-891 were dissolved in 0.5% methylcellulose (0.5% MC) at a concentration of 10 mg/mL, and gavage was administered at a volume of 10 mL/kg. Blood samples were collected over a 24 h period post-dose into tubes containing EDTA-K_2_, and plasma was separated with centrifugation at 5.645 g for 10 min. Plasma was collected and precipitated using two volumes of acetonitrile containing an internal standard. This step was followed by centrifugation at 15.680 g for 10 min after vortexing for 5 min. The supernatant was diluted with acetonitrile, and 10 µL of the supernatant was analyzed by Waters LC-PDA-MS/MS to determine plasma drug levels. Pharmacokinetic parameters were determined using mean data from four mice at each time point. Statistical analysis of the data was performed using the DAS 2.1.1 statistical software program (BioVoice, Shanghai, China).

### 4.3. Molecular Docking Study

The molecular docking experiment was performed using the CDOCKER docking algorithm of Discovery Studio 2020 (DS 2020). Compound **10f** and TUG-891 were drawn in ChemDraw Professional 16.0 and imported into Discovery Studio. Compounds were prepared to generate 3D conformations, tautomers, isomers, and appropriate ionization states at pH 7. Full ligand energy minimization was performed under the general-purpose all-atom forcefield CHARMm. The protein crystal structure of FFAR4 (PDB code 8G59) was downloaded from the Protein Data Bank (https://www.rcsb.org/, accessed on 2 December 2023). To prepare the protein structure for docking, water molecules and co-crystallized ligands were deleted, and the protein structures were then prepared by the Prepare Protein protocol using the DS 2020. The binding sites used to dock compounds represented the co-crystalized ligand, and all other parameters were maintained as defaults for the docking. Thirty docked poses of each compound were preserved, and the optimal docked orientation of each compound was chosen based on the docking score and maximal binding interactions.

## 5. Conclusions

DM is one of the most common metabolic diseases that severely threatens human health. An increasing number of studies suggested that FFAR4 may be a potential therapeutic target for metabolic diseases such as DM and obesity. To date, a large number of FFAR4 agonists were reported, although none of the FFAR4 agonists entered clinical trials. Therefore, significant efforts are underway to develop novel FFAR4 agonists with improved efficacy and metabolic stability. In the present study, based on the structure of TUG-891, which has excellent activity and selectivity, a series of novel FFAR4 agonists was designed by replacing the phenylpropanoic acid β-position carbon atom with an oxygen atom, while replacing the linking oxymethylene with an amide-linking group. The target compounds were synthesized, and the structures of all target compounds were characterized by ^1^H NMR and ^13^C NMR and confirmed by HRMS. Compounds **1f**–**12f** were studied for their FFAR4 agonist activity in vitro, and the results showed that compounds **5f**, **10f**, and **11f** exhibited excellent FFAR4 agonistic activity and higher hydrophilicity than TUG-891. Subsequent OGTTs on compounds **5f**, **10f**, and **11f** in normal ICR mice showed that compound **10f** exhibited the greatest improvement in glucose tolerance in normal ICR mice. Further antidiabetic activity studies in DIO mice showed that compound **10f** dose dependently decreased blood glucose levels and increased insulin secretion in DIO mice. Molecular docking analysis indicated that compound **10f** interacted with FFAR4 in a manner similar to that of TUG-891. These results indicate that compound **10f** is a promising compound that deserves further structure–activity relationship and pharmacological studies for the development of antidiabetic drugs.

## Figures and Tables

**Table 1:** In vitro activities of target compounds **1f**–**12f**.

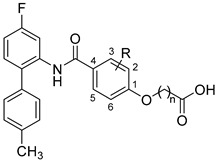					
Compound	n	R	CLogP ^a^	TPSA/Å^2^	hFFAR4 ^b^ (EC_50_, nM)
TUG-891	–	–	4.96	46.53	53.6
**1f**	1	H	4.06	75.63	135.6
**2f**	3	H	4.58	75.63	189.3
**3f**	4	H	4.93	75.63	235.6
**4f**	1	2-CH_3_	4.40	75.63	145.1
**5f**	3	2-CH_3_	4.94	75.63	112.6
**6f**	4	2-CH_3_	5.25	75.63	218.2
**7f**	1	3-CH_3_	4.42	75.63	189.1
**8f**	3	3-CH_3_	4.92	75.63	246.3
**9f**	4	3-CH_3_	5.15	75.63	278.6
**10f**	1	F	4.35	75.63	78.6
**11f**	3	F	4.91	75.63	93.2
**12f**	4	F	5.23	75.63	127.2

^a^ cLogP values and topological polar surface area (TPSA) were calculated using the online tool SwissADME available at http://www.swissadme.ch (accessed on 7 August 2024). ^b^ EC_50_ values for FFAR4 agonistic activities represent the mean of three determinations.

**Table 2:** Pharmacokinetic profiles of compound **10f** in ICR mice ^a^.

Compound	Dose (mg/kg)	*C*_max_ (µg/mL)	*T*_max_ (min)	AUC (µg·h/mL)	*T*_1/2_ (h)
TUG-891	10	1.24 ± 0.17	30	2.40 ± 0.25	0.74 ± 0.29
**10f**	10	3.14 ± 1.15 *	30	9.83 ± 2.93 **	1.51 ± 0.51 *

^a^ Values are expressed as the mean *±* SD of four ICR mice. TUG-891 and compound **10f** were suspended in a 0.5% methyl cellulose aqueous solution. AUC, area under the curve; *C*_max_, the maximum drug concentration in plasma; *T*_max_, the time to reach maximum drug concentration in plasma; *T*_1/2_, half-life. * *p* ≤ 0.05 compared with TUG-891-treated group by Student’s *t* test; ** *p* ≤ 0.01 compared with TUG-891-treated group by Student’s *t* test.

## Data Availability

The data presented in this study are available upon request from the corresponding author.

## Supplementary Materials

The following supporting information can be downloaded at: https://www.mdpi.com/article/10.3390/ijms252111476/s1.
