# Supplementary material for: Novel Phenoxyalkanoic Acid Derivatives as Free Fatty Acid Receptor 4 Agonists for Treating Type 2 Diabetes Mellitus
Source: Int J Mol Sci. 2024 Oct 25;25(21):11476. doi: 10.3390/ijms252111476 (PMC11547093; doi:10.3390/ijms252111476)
Supplement: Supplementary file 1 [file ijms-25-11476-s001.zip › ijms-3223760-supplementary.pdf]

Novel Phenoxyalkanoic Acid Derivatives as Free Fatty Acid Receptor 4 Agonists for Treating Type 2 Diabetes Melli-tus

Xu Li [1], Xinmeng Zhang [1], Xueyuan Xie [1], Taimin Dong [1], Chengxu Lv [1], Ranran Guan [1], Wenyue Zhang [1], Guoxia Ji [2], Fanghui, Chen [1], Shiben Wang [1,*] and Xuekun Wang [1,*]

Contents of SI

Section	Contents	page
1	Experimental procedure of FFAR4 and FFAR1Agonistic Activity	S2
2	Interaction of TUG-891 with free fatty acid receptor 4	S2
3	Oral Glucose Tolerance Test (OGTT) of TUG-891 in Normal ICR Mice	S3
4	The NMR, HRMS and HPLC spectra of compounds **1f–12f**	S4-27

1. In Vitro FFAR4 Agonistic Activity Studies

A calcium flux assay was performed to assess the agonist activity of the test compounds against the hFFAR4 receptor. CHO cells stably transfected with hFFAR4 were seeded in a 96-well plate at a density of 20,000 cells per well and incubated for 24 h at 37 °C in a 5% CO_2 incubator. The medium in the wells was gently discarded, and the wells were washed with Hank's balanced salt solution (HBSS; 100 μL per well). Cells were then incubated in HBSS containing Fluo-4 AM (2.5 μg/mL), 0.1% fatty acid-free bovine serum albumin, and probenecid (2.5 mM) for 60 min at 37 °C. The cells were then washed thrice in HBSS and allowed to equilibrate for 10 min before conducting the assay. Test compounds dissolved in dimethyl sulfoxide were diluted in HBSS at various concentrations and added to Fluo-4 AM-containing cells, and intracellular Ca^{2+} concentrations were measured using FlexStation 3 Molecular Devices. The EC_{50} value of each compound was calculated using GraphPad Prism software (version 5.0; GraphPad Software, San Diego, CA, USA). The calcium influx assay of compound **10f** in hFFAR1-expressing CHO cells was similar to that of hFFAR4

2. Interaction of TUG-891 with free fatty acid receptor 4

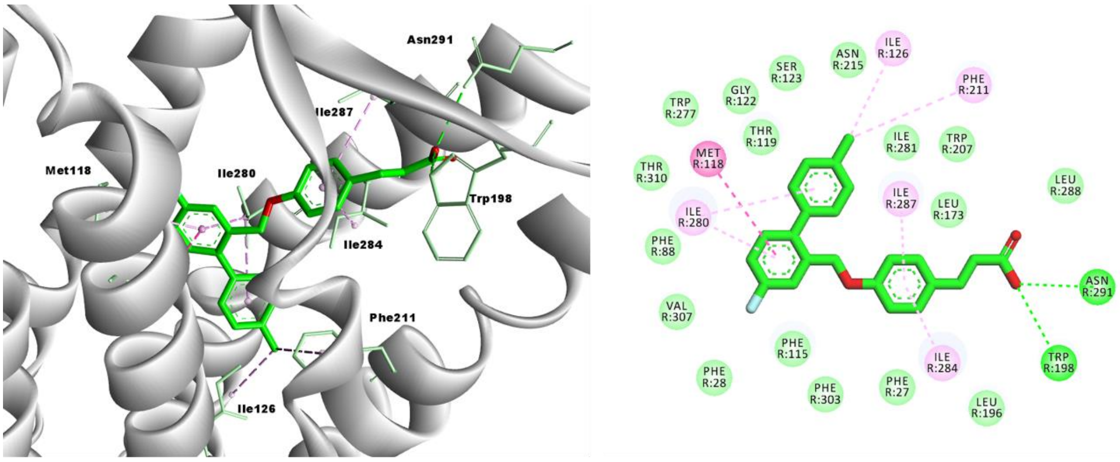

(a) (b)

Figure S1. Interaction of TUG-891 with free fatty acid receptor 4 (PDB code 8G59). (a) 3D pose of TUG-891 with free fatty acid receptor 4 (protein is shown in gray, compound **10f** in blue and amino acid residues in green), (b) 2D plot of TUG-891 with free fatty acid receptor 4 (green circles and lines represent conventional hydrogen bonds, pink circles and lines represent π–π and π–alkyl interactions).

3. Oral Glucose Tolerance Test (OGTT) of TUG-891 in Normal ICR Mice

Male ICR mice, four-week aged, were purchased from Jinan Pengyue Experimental Animal Breeding Co., Ltd. (Jinan, China). The mice were housed in cages under a 12 h light/dark cycle from 7:00 to 19:00 at controlled temperatures (25–26 °C) and relative humidity (50 ± 10%) throughout the experimental period. All animals were allowed to eat and drink freely unless otherwise stated, and were allowed to acclimatize for 1 week before the experiment. All animal experimental protocols were performed following applicable institutional and governmental regulations concerning the ethical use of animals.

Normal male ICR mice were weighed and randomly divided into six groups (six mice per group) after 12 h of fasting. TUG-891 was dissolved in 0.5% MC and vortexed before the study initiation. Mice in each group were gavaged with vehicle (0.5% MC aqueous solution, 10 mL/kg), or TUG-891(1, 3, 10, 30 mg/kg; 10 mL/kg), 30 min before oral glucose loading (2 g/kg, 10 mL/kg). The exact dose volume was calculated separately for each animal. Blood samples were collected via the tail tip 30 min before the compound dose, at t = 0 (immediately before glucose loading), and 15, 30, 60, and 120 min after glucose loading. Blood glucose levels were measured using blood glucose test strips (Sannuo GA-3 type; Changsha, China). Glucose values were entered into an Excel sheet and plotted using GraphPad Prism software.

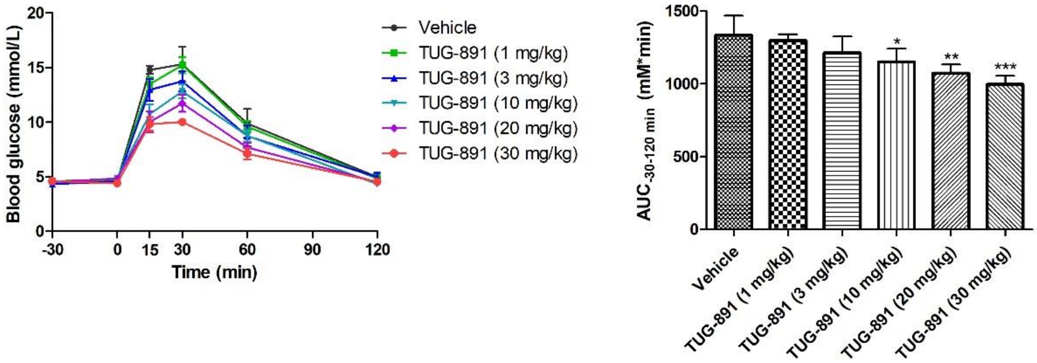

Figure S2. Effects of compound TUG-891 on plasma glucose levels during the oral glucose tolerance test in fasting ICR mice. (a) Time-dependent changes of blood glucose levels after oral administration of TUG-891, followed by the oral glucose load (2 g/kg). (b) $AUC_{-30–120\ min}$ of plasma glucose levels. Values are expressed as the mean ± SD (n = 6). * $p \leq 0.05$ compared with vehicle-treated group by Student's t test; ** $p \leq 0.01$ compared with vehicle-treated group by Student's t test; *** $p \leq 0.001$ compared with vehicle-treated group by Student's t test.

4. The NMR, HRMS and HPLC spectra of compounds 1f–12f

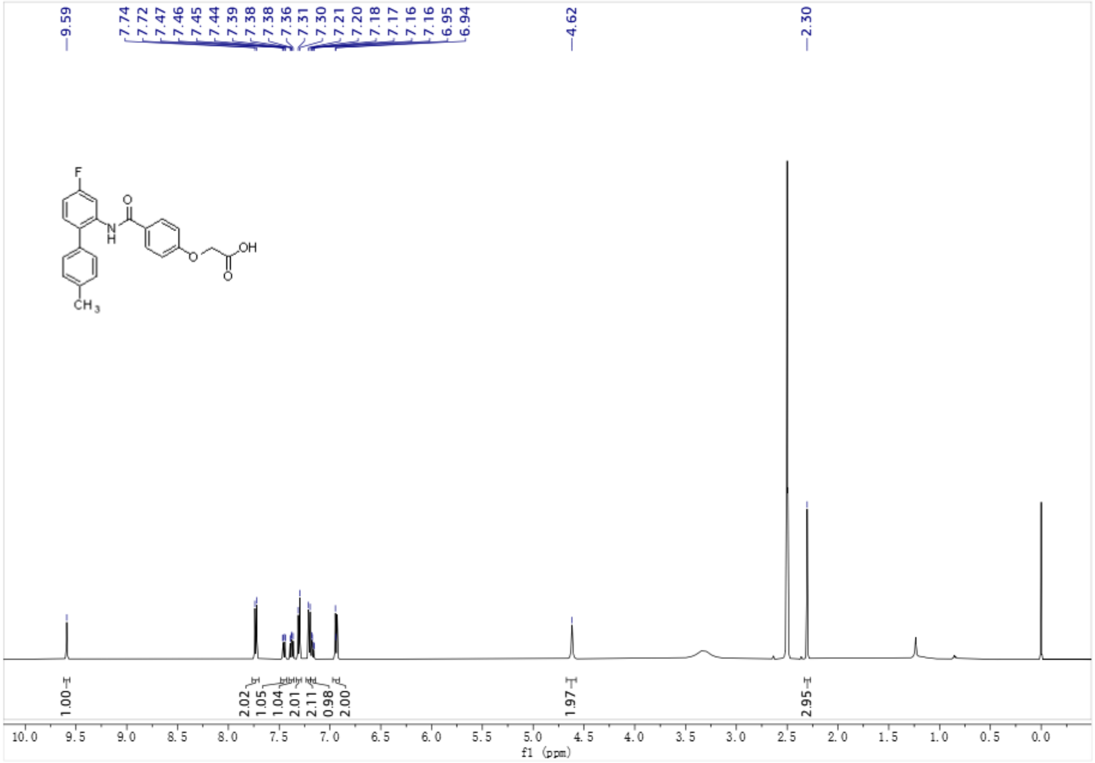

Figure S3. ^{1}H NMR spectrum for **1f** (DMSO-*d6*, 500 MHz)

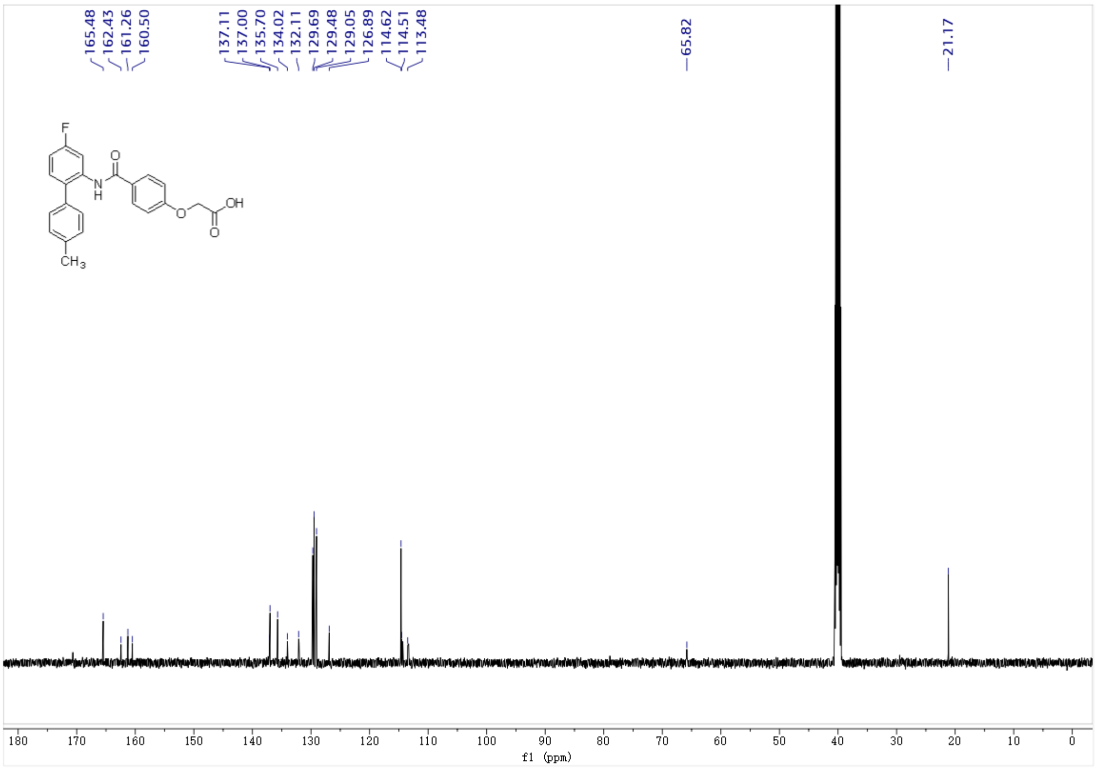

Figure S4. ^{13}C NMR spectrum for **1f** (DMSO-*d6*, 125 MHz)

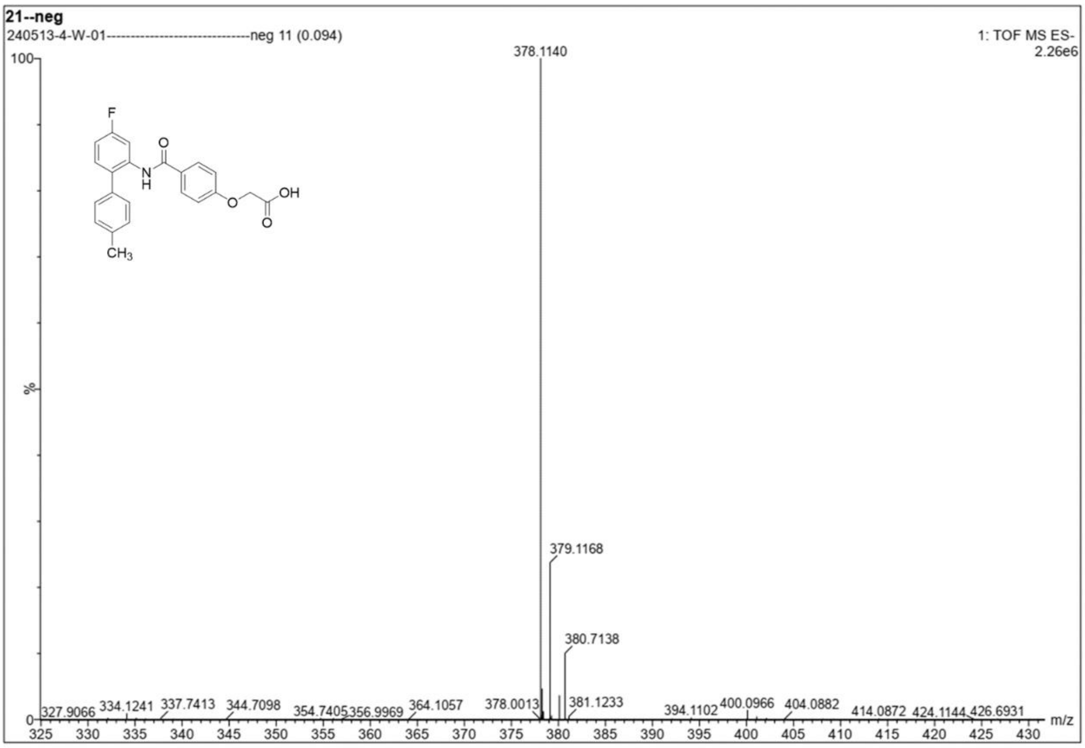

Figure S5. HRMS spectra of compound **1f**

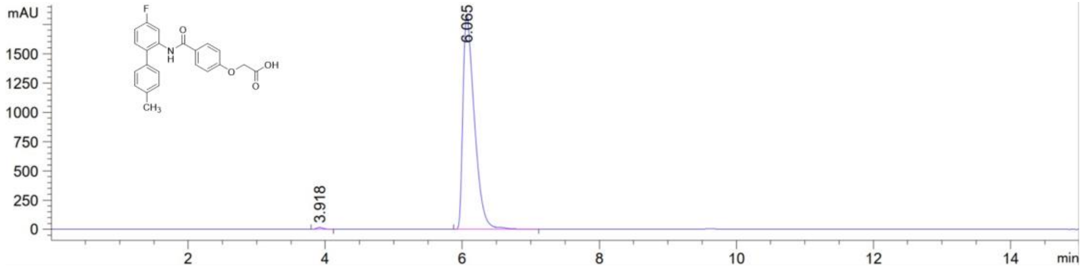

Peak #	Retention time [min]	Type	Peak width [min]	Peak area [mAU*s]	Peak height [mAU]	Peak area ratio %
1	3.918	BB	0.1016	103.83814	15.61286	0.4776
2	6.065	BB	0.1822	2.16383e4	1826.68616	99.5224

Figure S6. HPLC spectra of compound **1f**

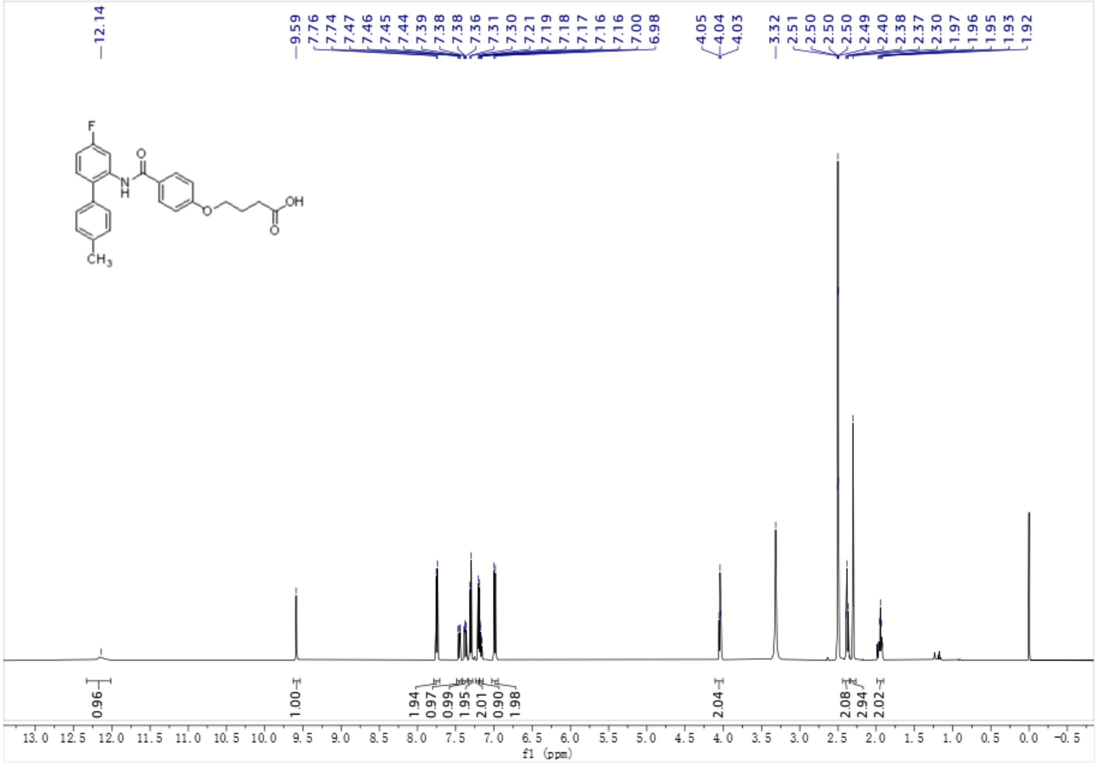

Figure S7. ^{1}H NMR spectrum for **2f** (DMSO-*d6*, 500 MHz)

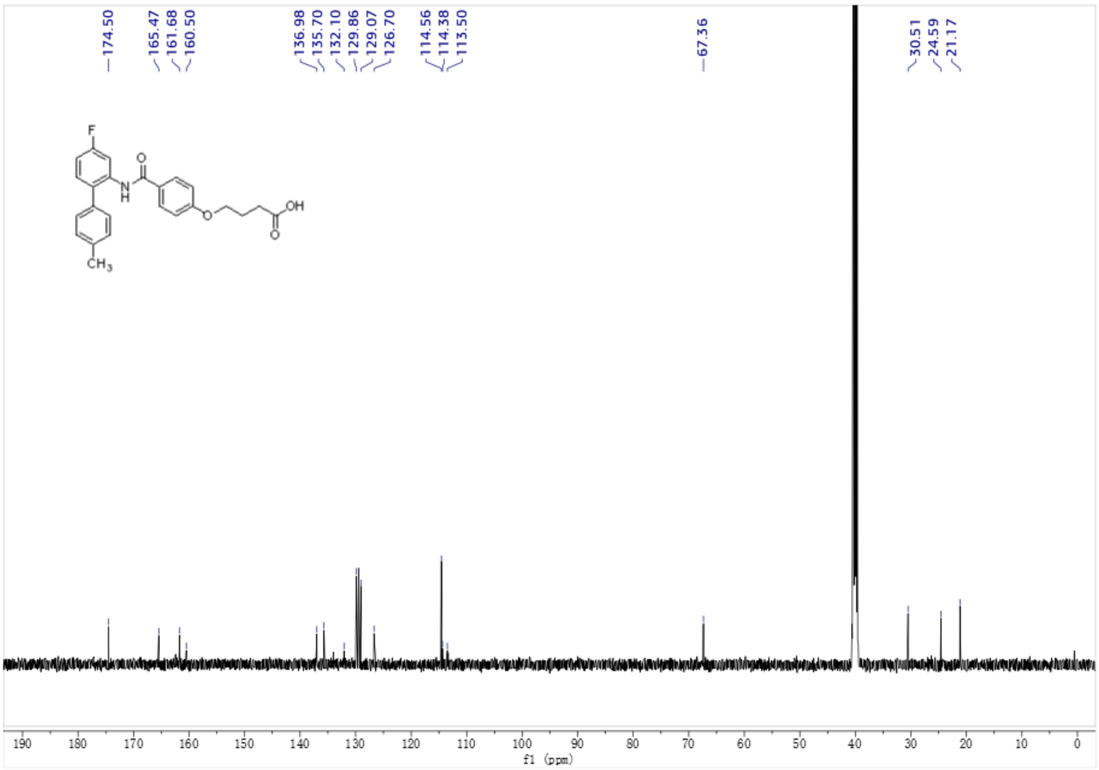

Figure S8. ^{13}C NMR spectrum for **2f** (DMSO-*d6*, 125 MHz)

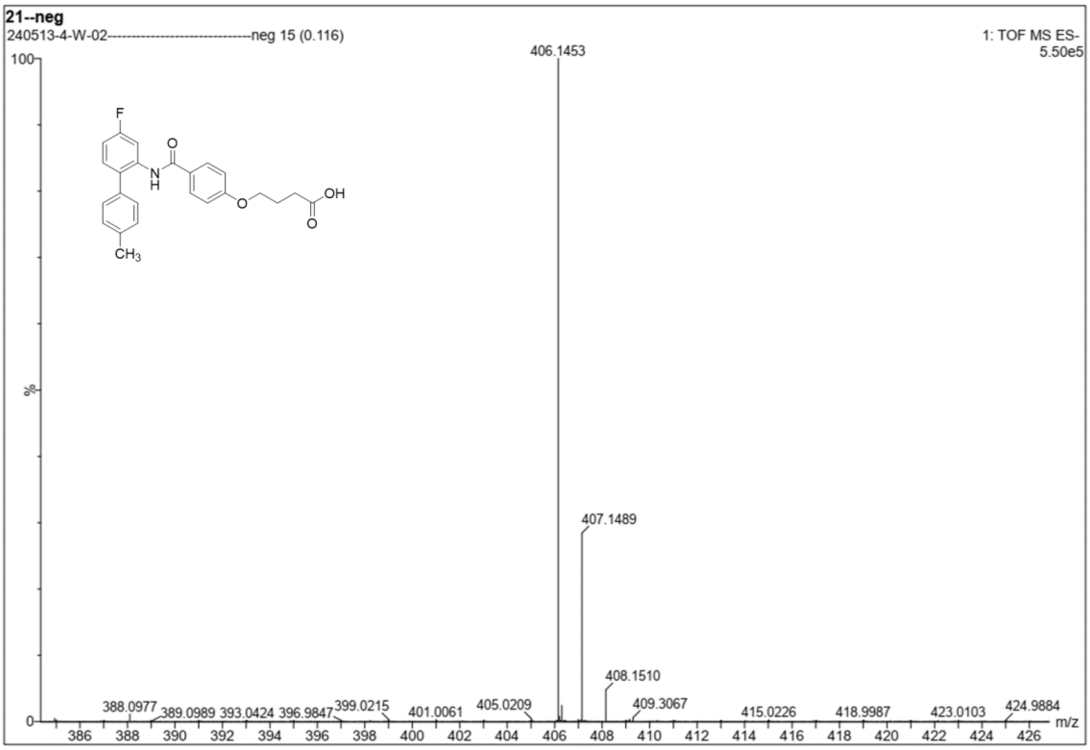

Figure S9. HRMS spectra of compound **2f**

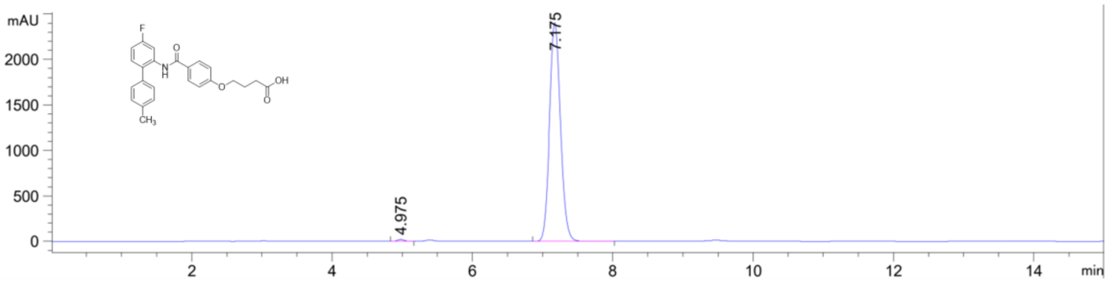

Peak #	Retention time [min]	Type	Peak width [min]	Peak area [mAU*s]	Peak height [mAU]	Peak area ratio %
1	4.975	BB	0.1092	145.15361	20.85064	0.5539
2	7.175	BB	0.1686	2.60620e4	2401.39819	99.4461

Figure S10. HPLC spectra of compound **2f**

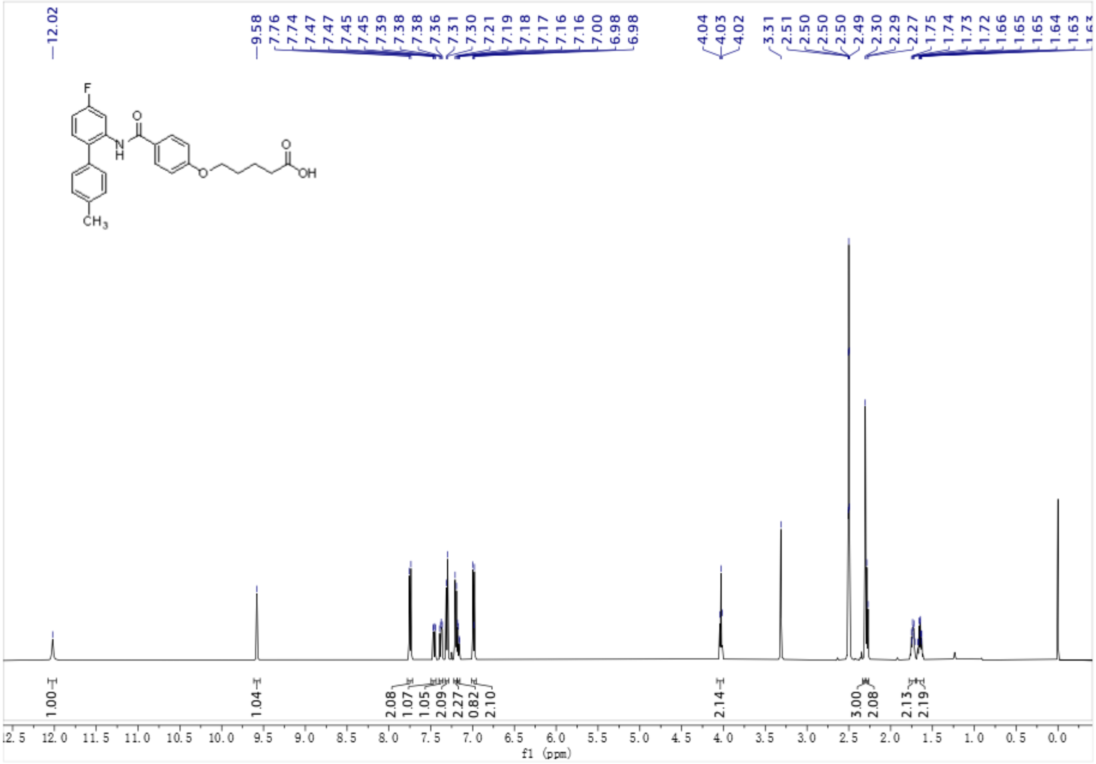

Figure S11. ^{1}H NMR spectrum for **3f** (DMSO-*d6*, 500 MHz)

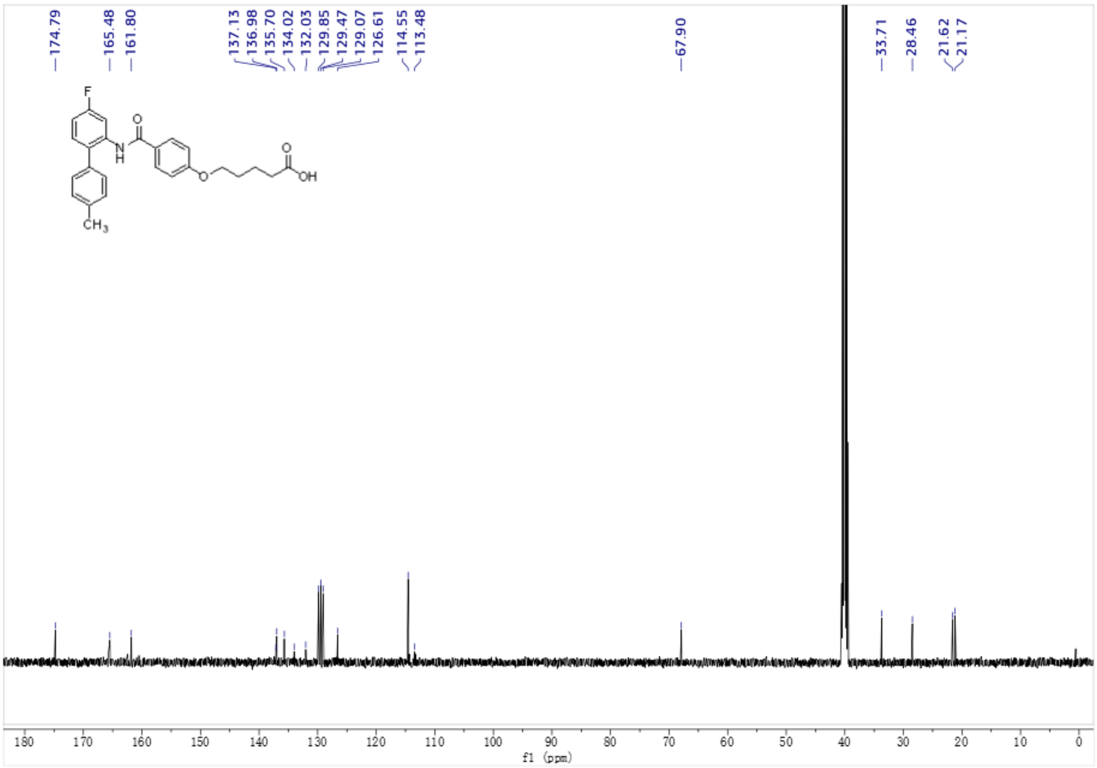

Figure S12. ^{13}C NMR spectrum for **3f** (DMSO-*d6*, 125 MHz)

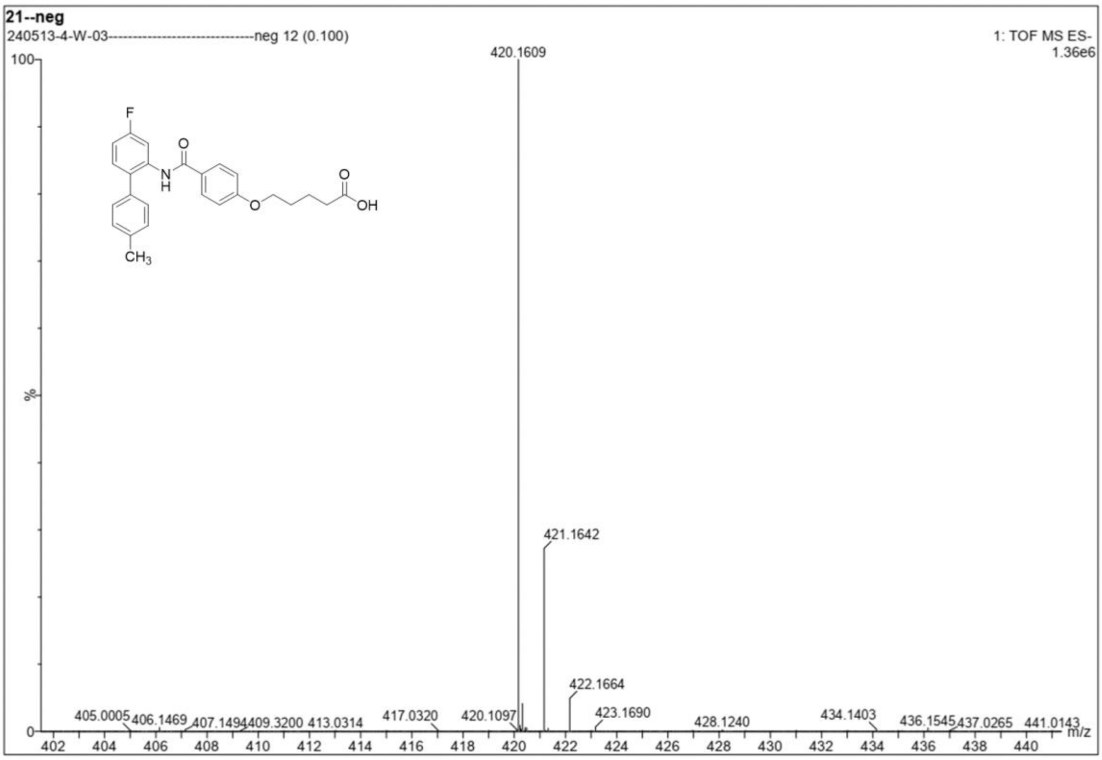

Figure S13. HRMS spectra of compound **3f**

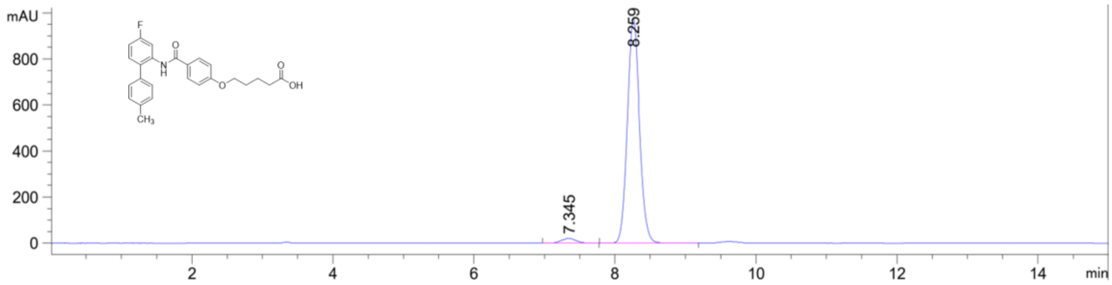

Peak #	Retention time [min]	Type	Peak width [min]	Peak area [mAU*s]	Peak height [mAU]	Peak area ratio %
1	7.345	BB	0.2290	287.72678	19.86291	2.4036
2	8.259	BB	0.1859	1.16828e4	974.36005	97.5964

Figure S14. HPLC spectra of compound **3f**

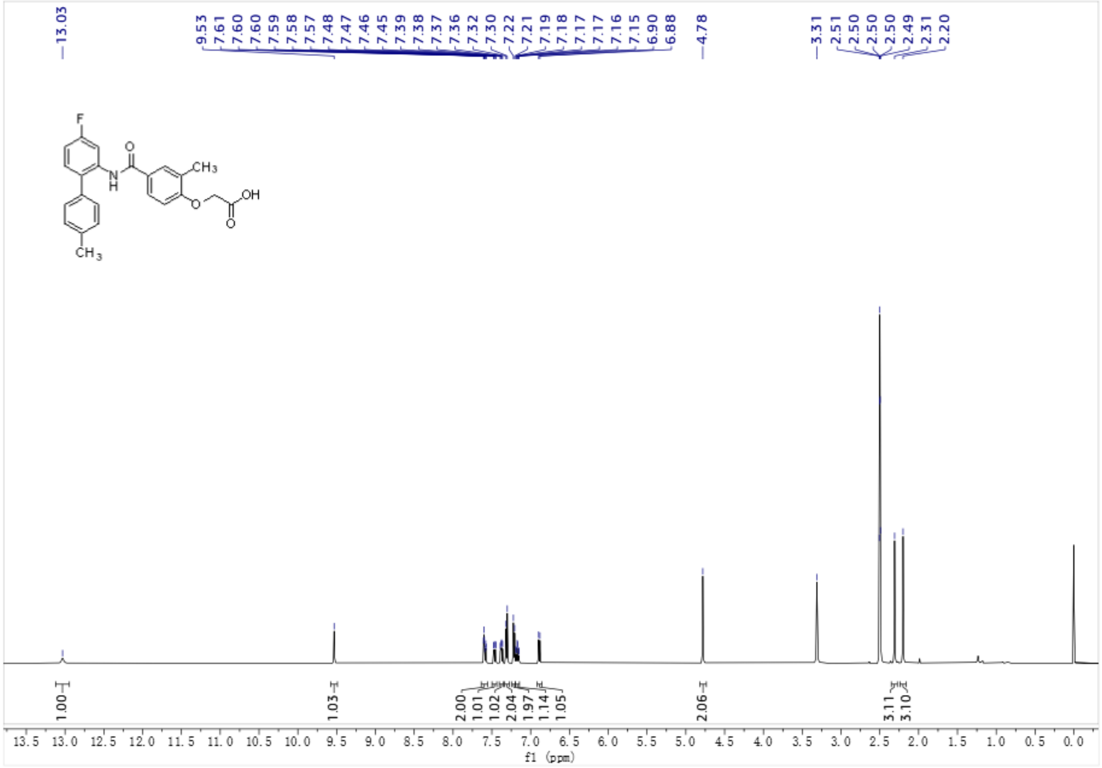

Figure S15. ^{1}H NMR spectrum for **4f** (DMSO-*d6*, 500 MHz)

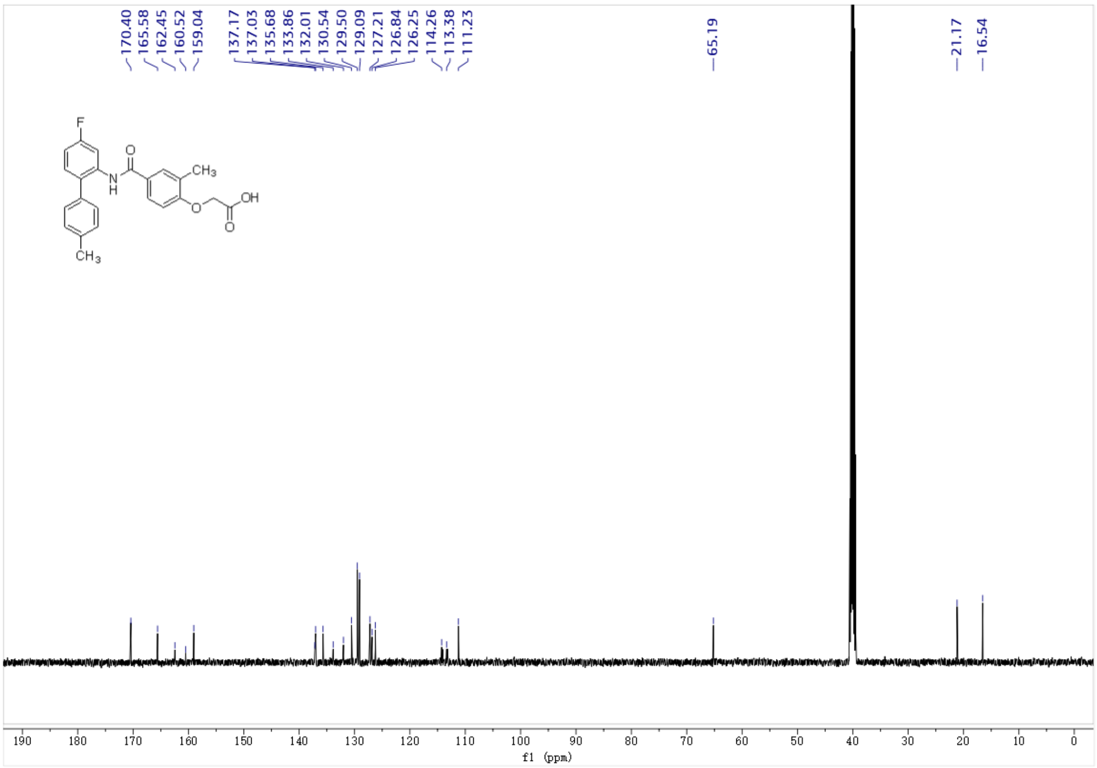

Figure S16. ^{13}C NMR spectrum for **4f** (DMSO-*d6*, 125 MHz)

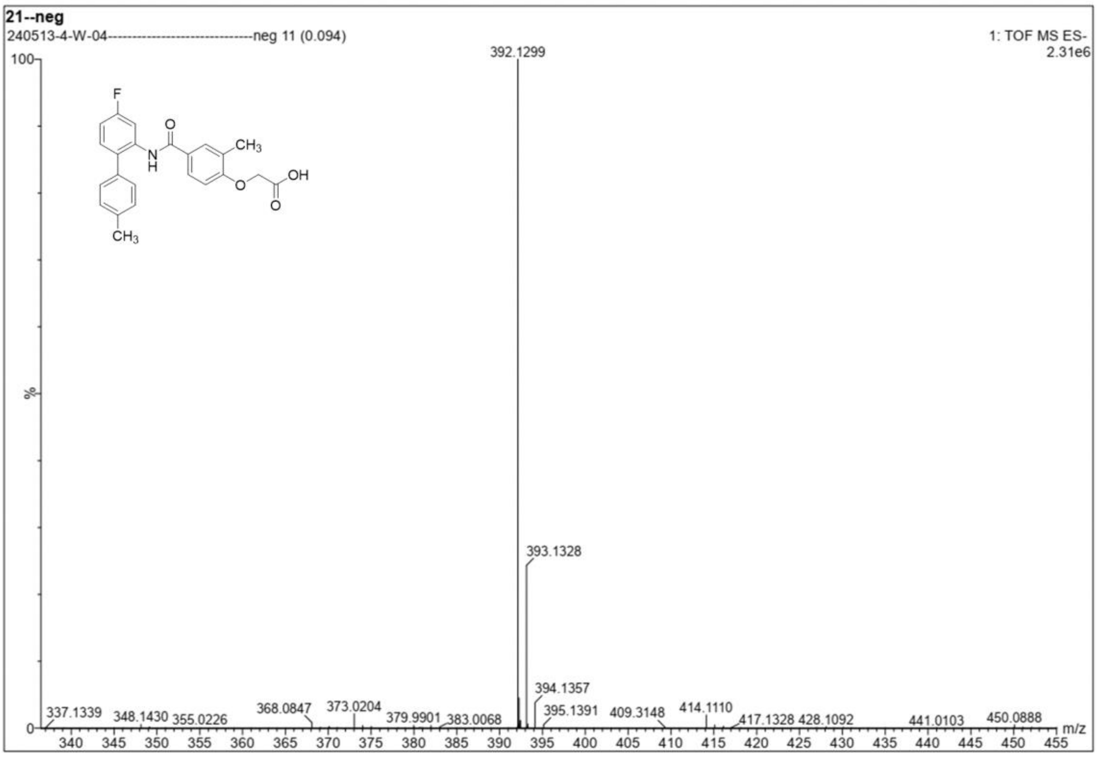

Figure S17. HRMS spectra of compound **4f**

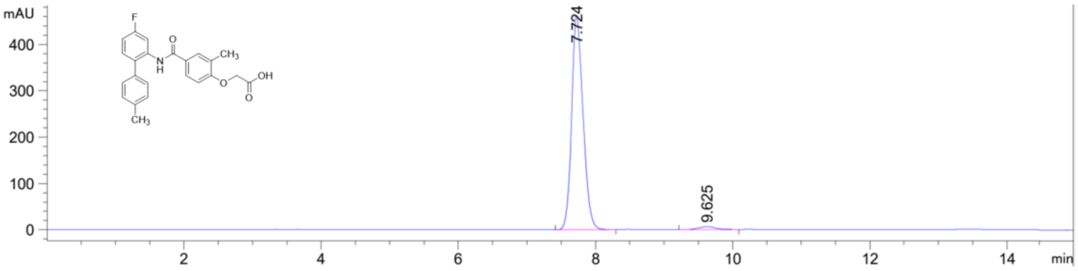

Peak #	Retention time [min]	Type	Peak width [min]	Peak area [mAU*s]	Peak height [mAU]	Peak area ratio %
1	7.724	BB	0.1810	5293.36914	457.30359	97.8449
2	9.625	BB	0.2772	116.58804	6.44169	2.1551

Figure S18. HPLC spectra of compound **4f**

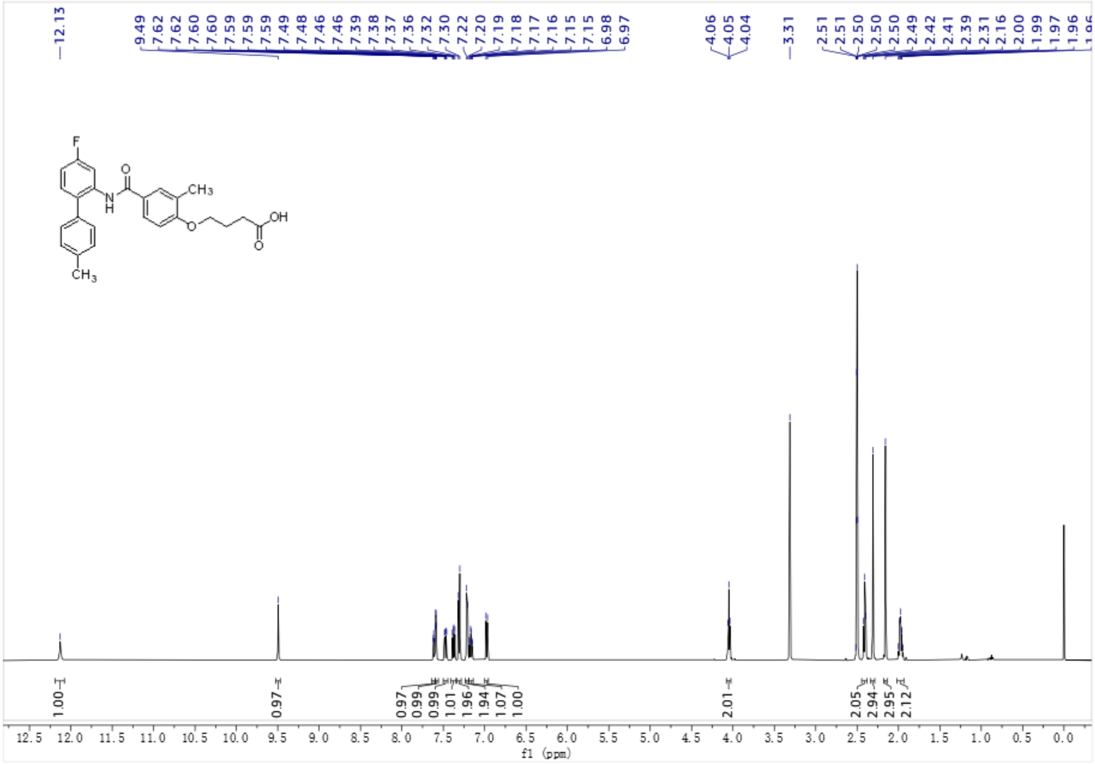

Figure S19. ^{1}H NMR spectrum for **5f** (DMSO-*d6*, 500 MHz)

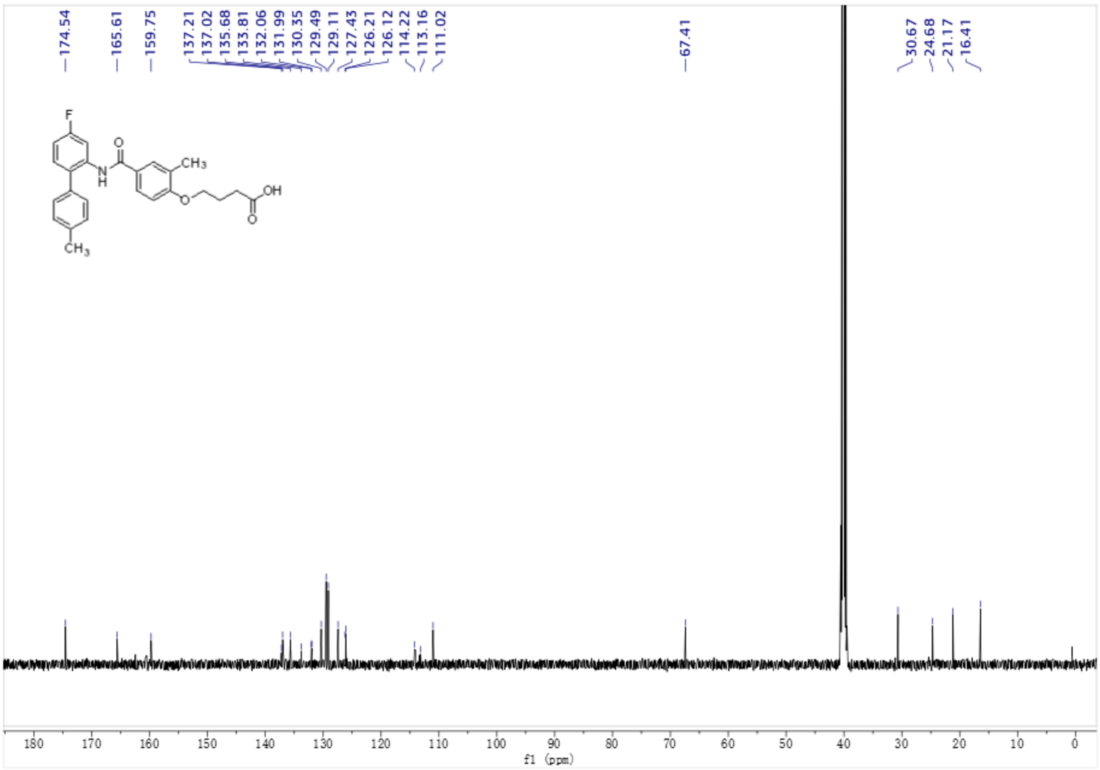

Figure S20. ^{13}C NMR spectrum for **5f** (DMSO-*d6*, 125 MHz)

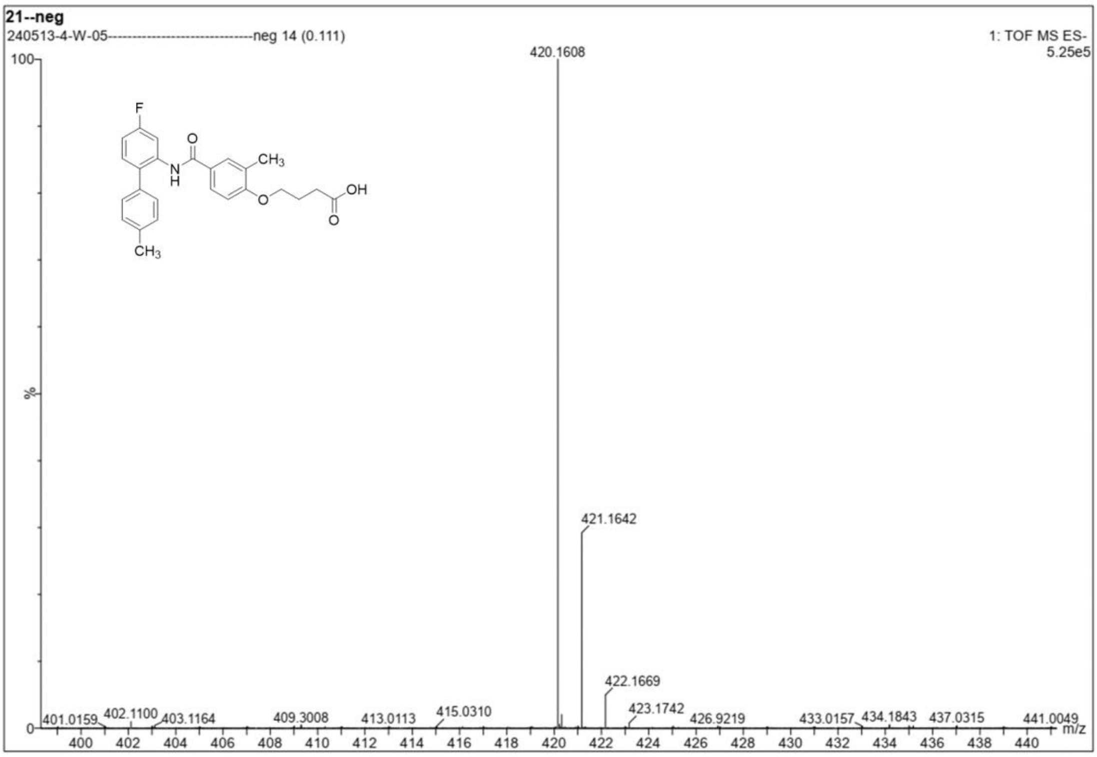

Figure S21. HRMS spectra of compound **5f**

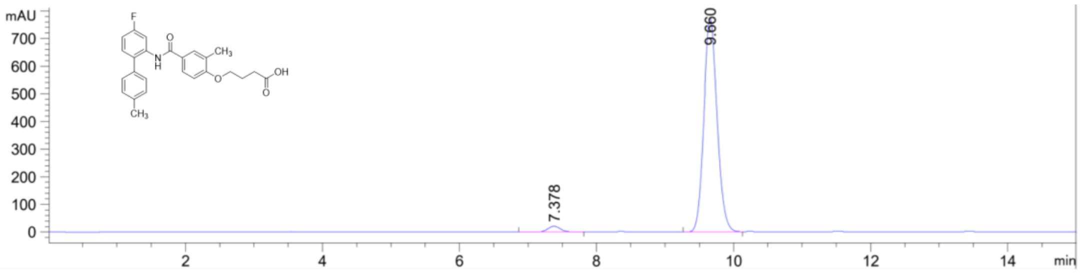

Peak	Retention time	Type	Peak width	Peak area	Peak height	Peak area ratio
#	[min]		[min]	[mAU*s]	[mAU]	%
1	7.378	BB	0.1987	268.31973	20.77044	2.5128
2	9.660	BB	0.2071	1.04099e4	773.00995	97.4872

Figure S22. HPLC spectra of compound **5f**

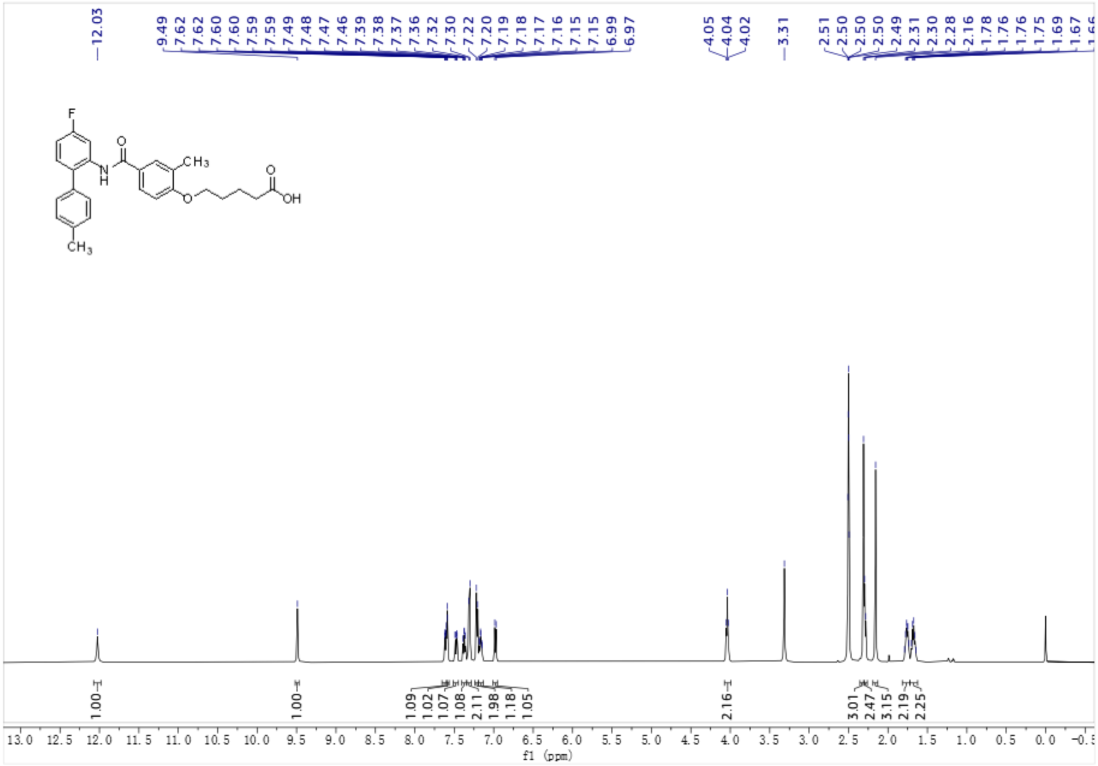

Figure S23. ^{1}H NMR spectrum for **6f** (DMSO-*d6*, 500 MHz)

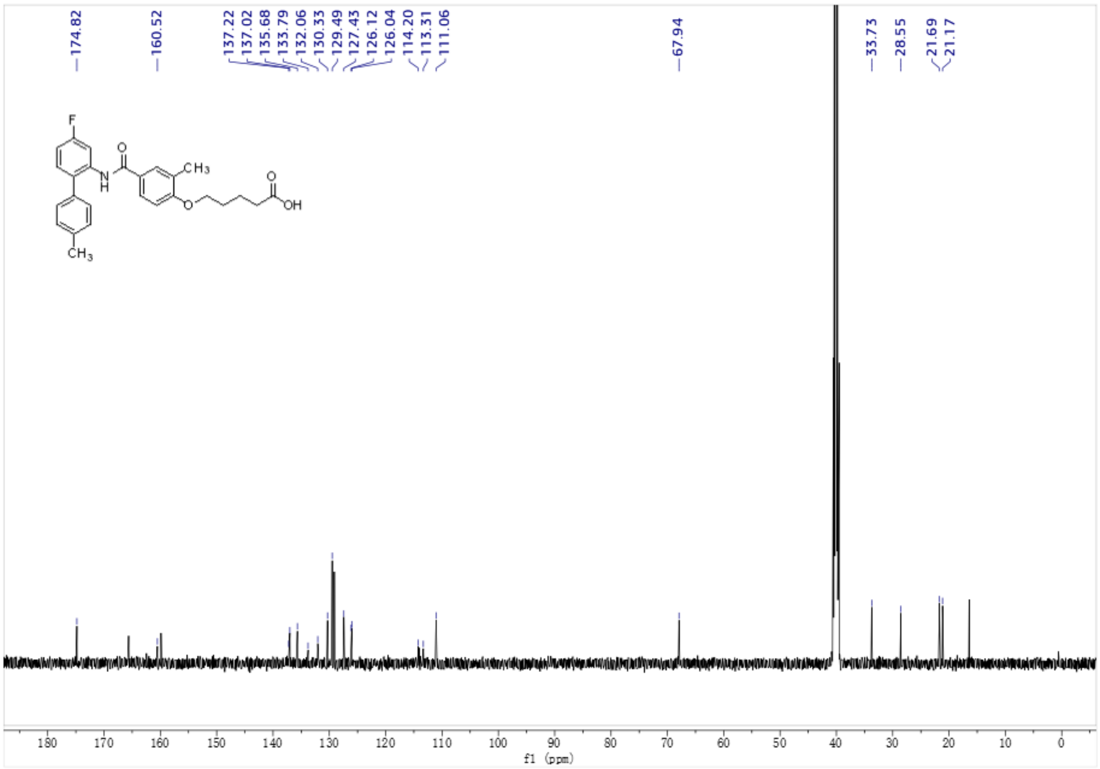

Figure S24. ^{13}C NMR spectrum for **6f** (DMSO-*d6*, 125 MHz)

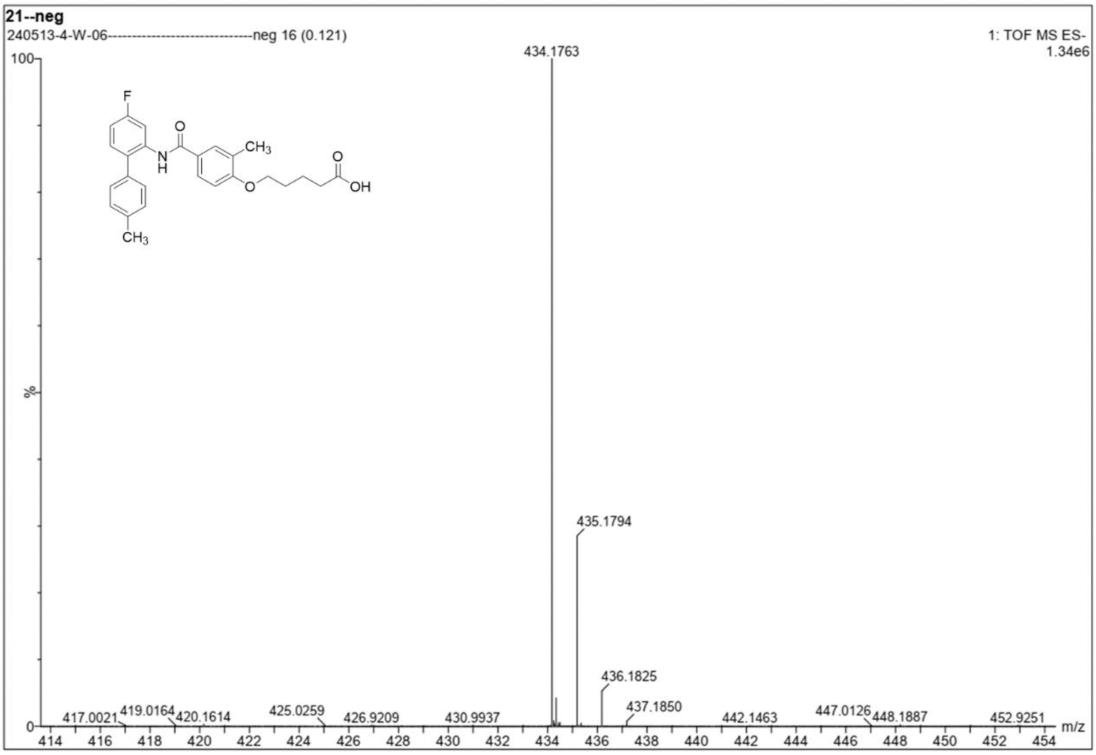

Figure S25. HRMS spectra of compound **6f**

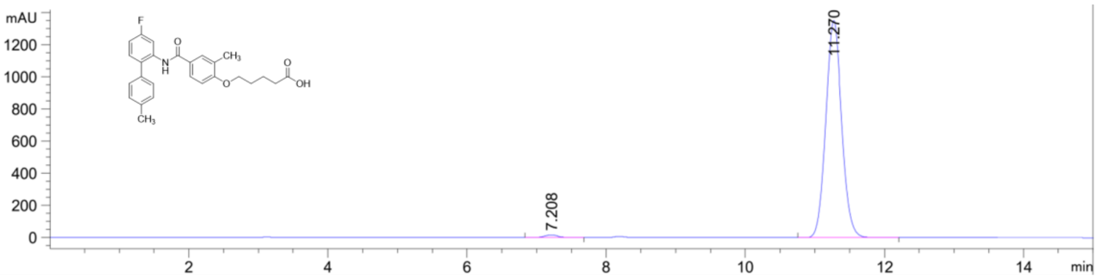

Peak #	Retention time [min]	Type	Peak width [min]	Peak area [mAU*s]	Peak height [mAU]	Peak area ratio %
1	7.208	BB	0.2047	227.00998	16.90449	1.0497
2	11.270	BB	0.2446	2.13989e4	1352.26599	98.9503

Figure S26. HPLC spectra of compound **6f**

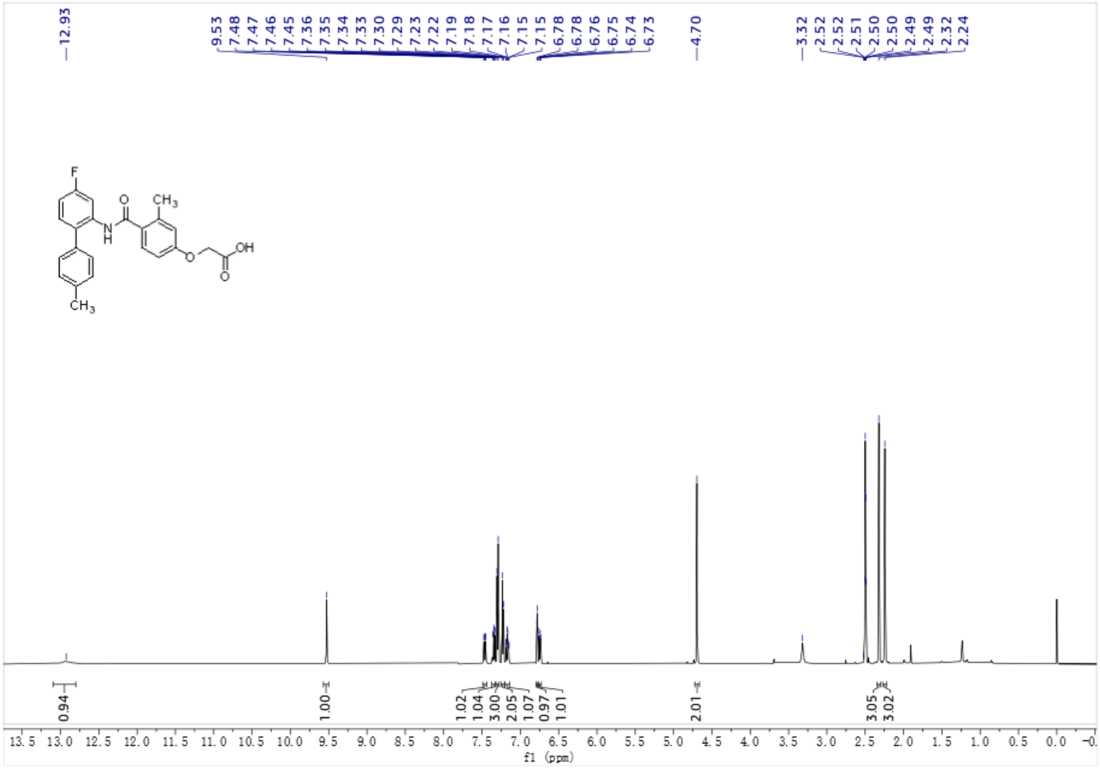

Figure S27. ^{1}H NMR spectrum for **7f** (DMSO-*d6*, 500 MHz)

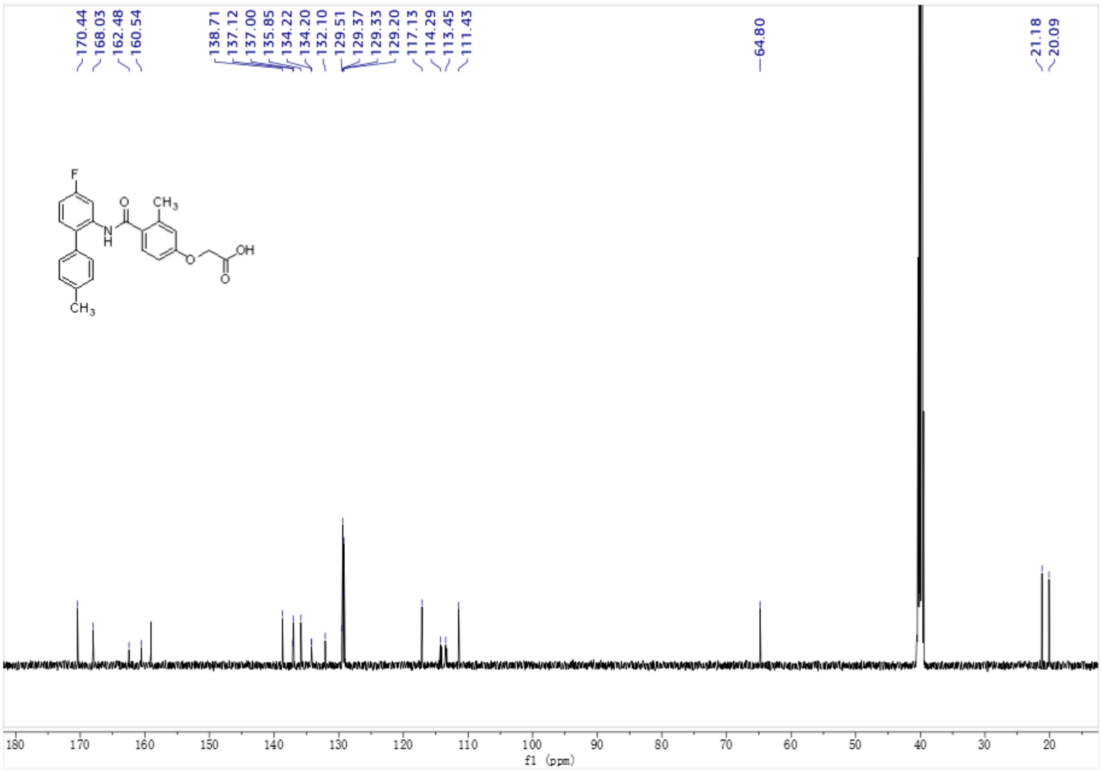

Figure S28. ^{13}C NMR spectrum for **7f** (DMSO-*d6*, 125 MHz)

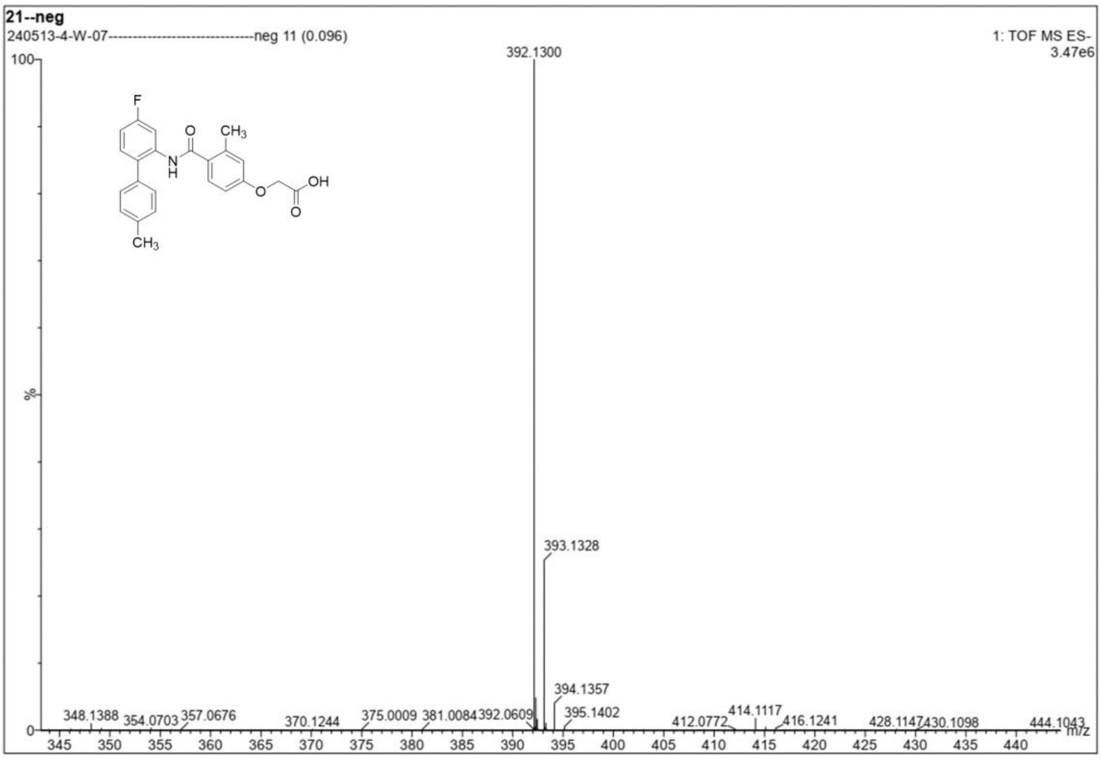

Figure S29. HRMS spectra of compound **7f**

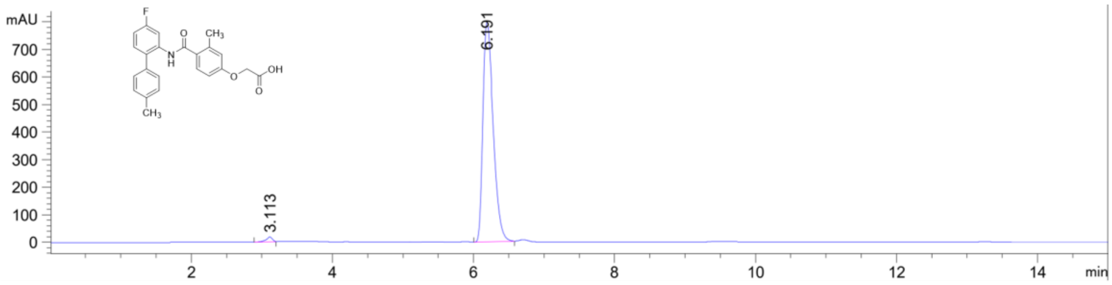

Peak #	Retention time [min]	Type	Peak width [min]	Peak area [mAU*s]	Peak height [mAU]	Peak area ratio %
1	3.113	BB	0.0870	105.45177	17.31775	1.2777
2	6.191	BB	0.1592	8147.62549	797.64990	98.7223

Figure S30. HPLC spectra of compound **7f**

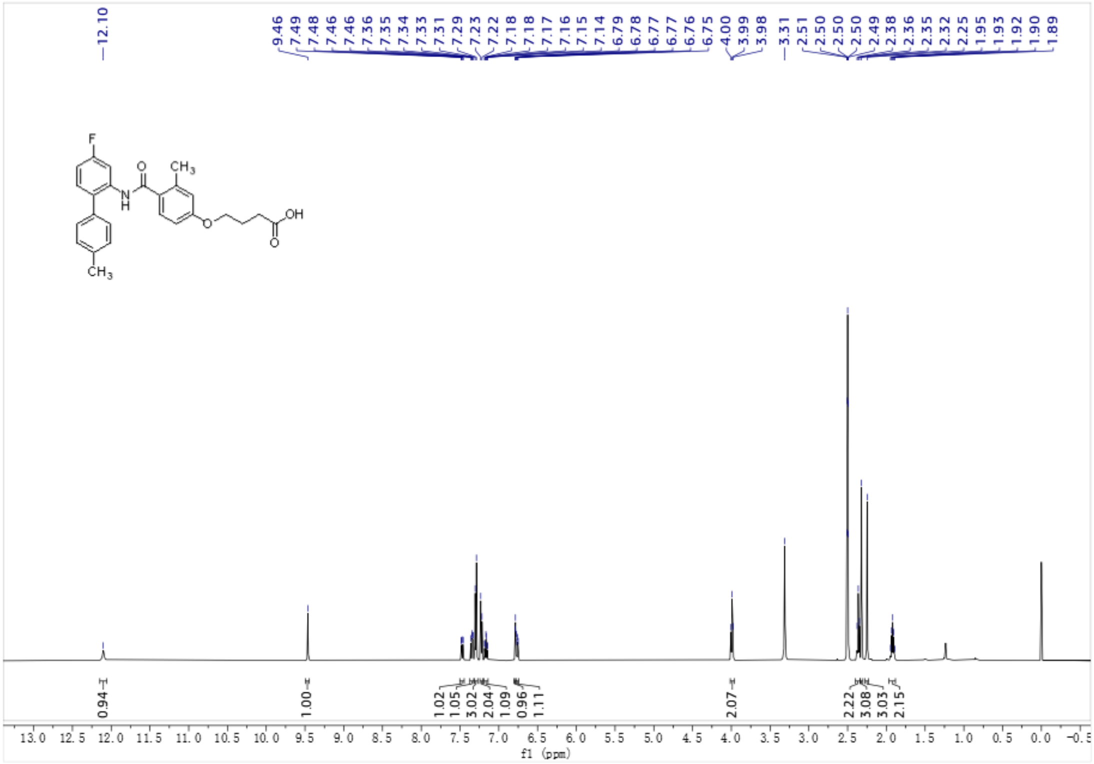

Figure S31. ^{1}H NMR spectrum for **8f** (DMSO-*d6*, 500 MHz)

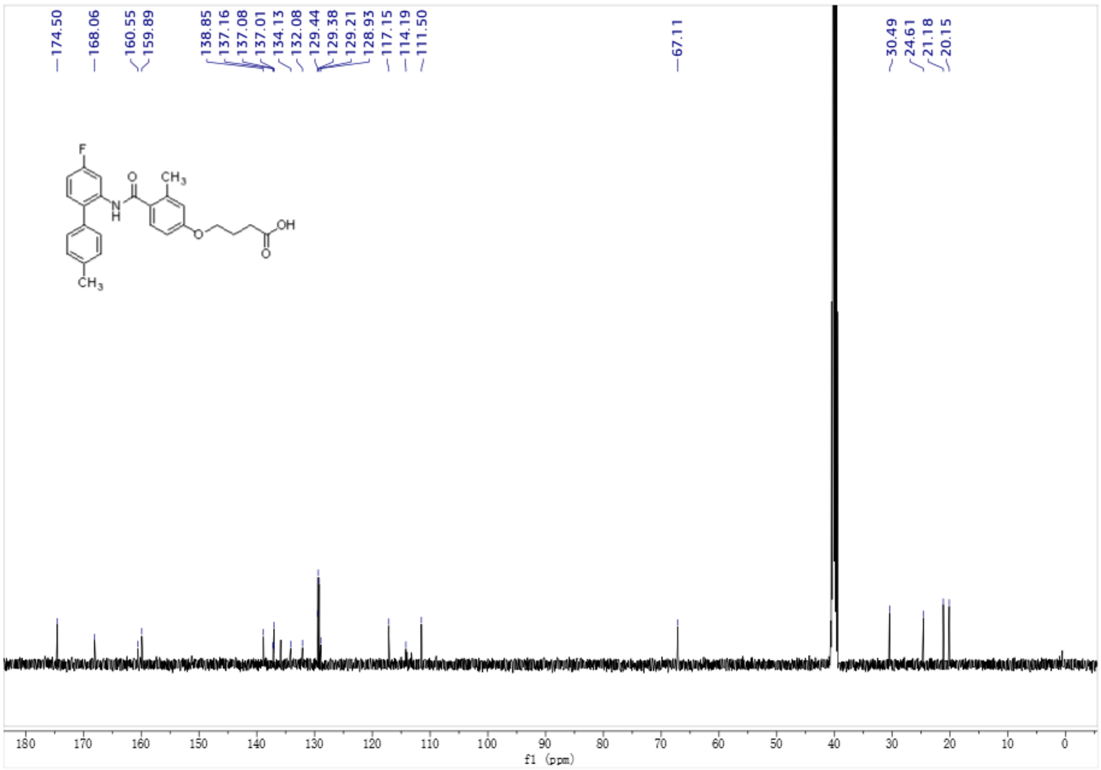

Figure S32. ^{13}C NMR spectrum for **8f** (DMSO-*d6*, 125 MHz)

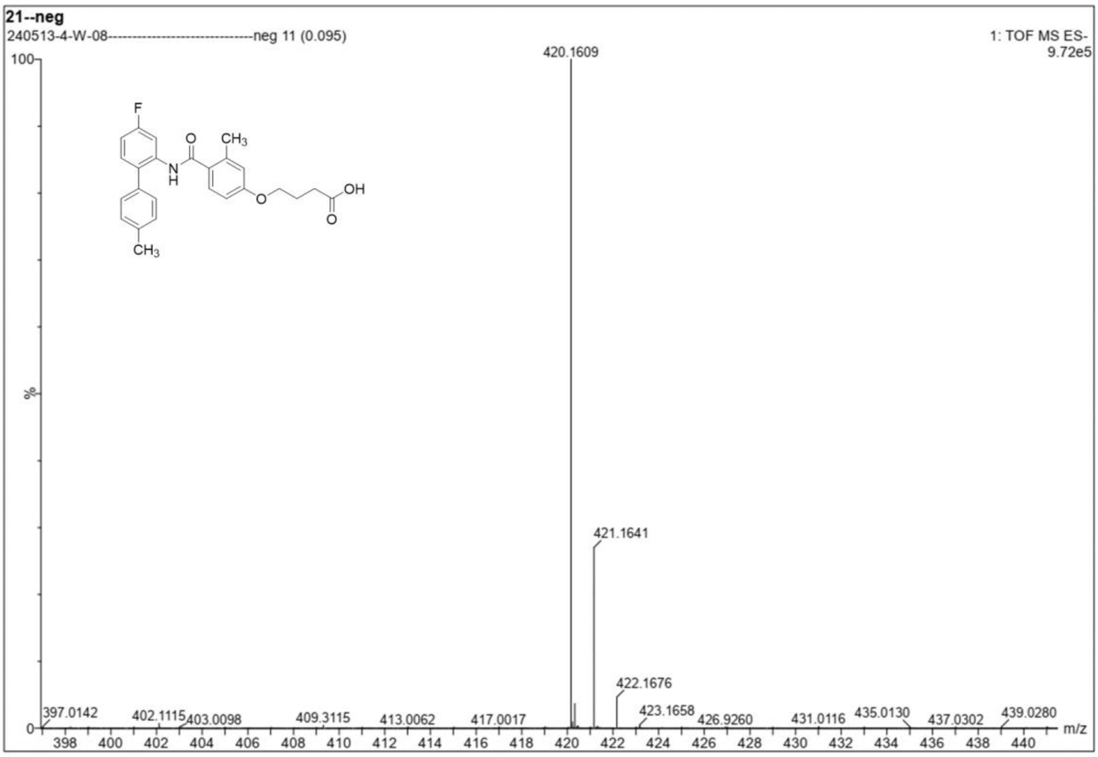

Figure S33. HRMS spectra of compound **8f**

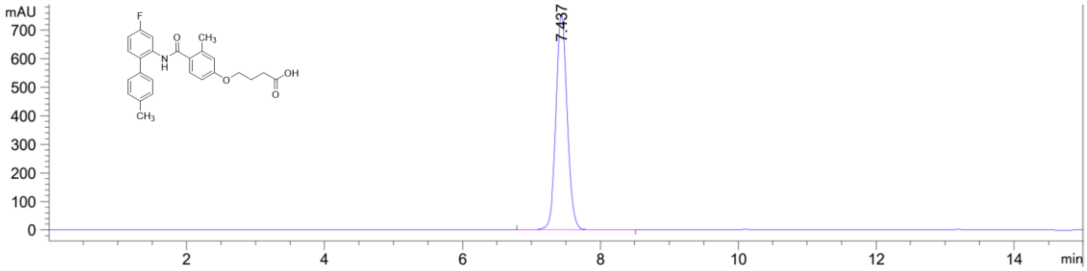

Peak	Retention time	Type	Peak width	Peak area	Peak height	Peak area ratio
#	[min]		[min]	[mAU*s]	[mAU]	%
1	7.437	BB	0.1731	8486.33398	755.44586	100.0000

Figure S34. HPLC spectra of compound **8f**

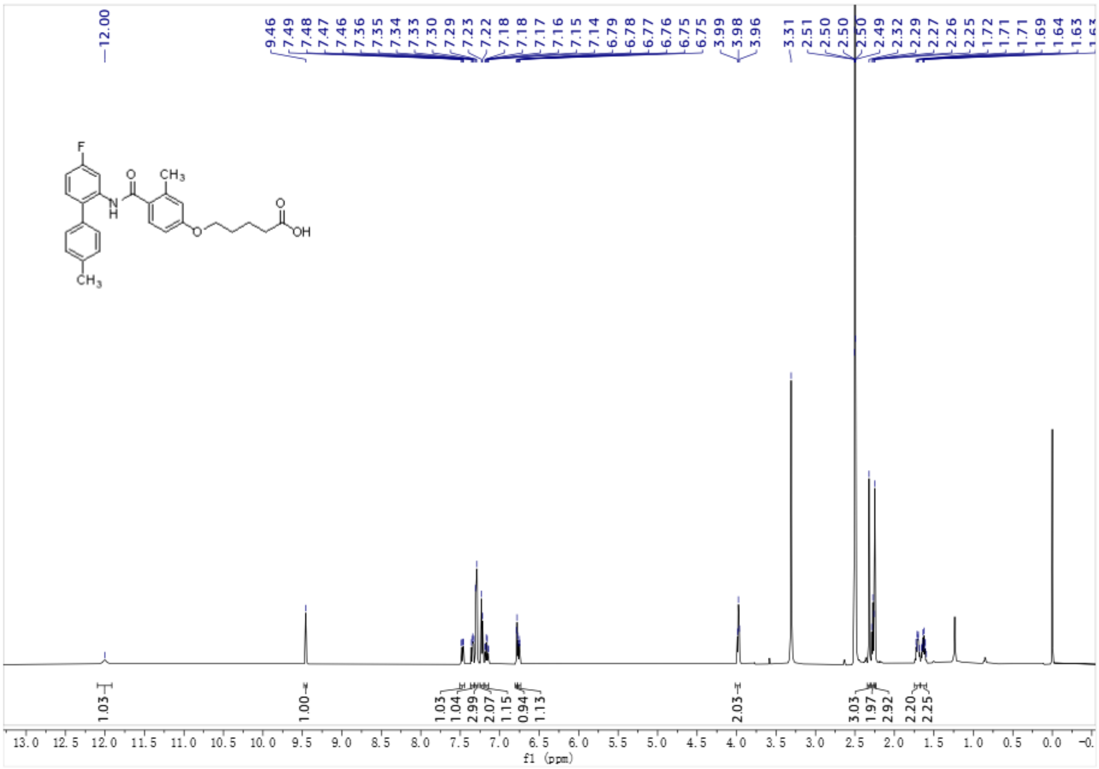

Figure S35. ¹H NMR spectrum for **9f** (DMSO-*d6*, 500 MHz)

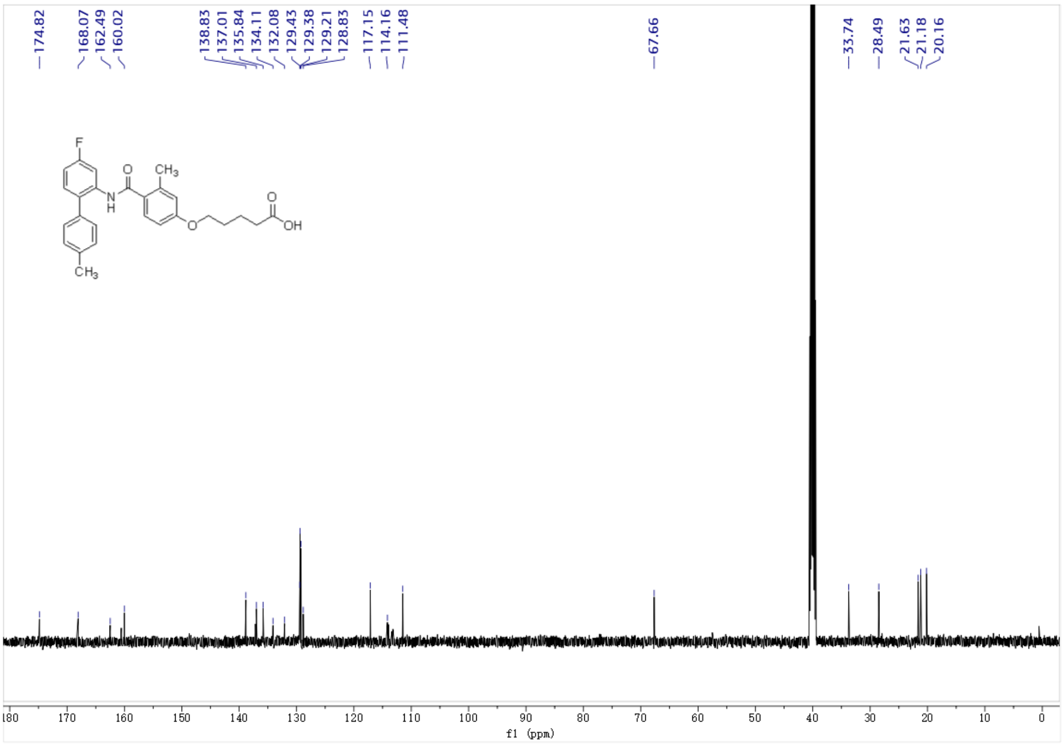

Figure S36. ¹³C NMR spectrum for **9f** (DMSO-*d6*, 125 MHz)

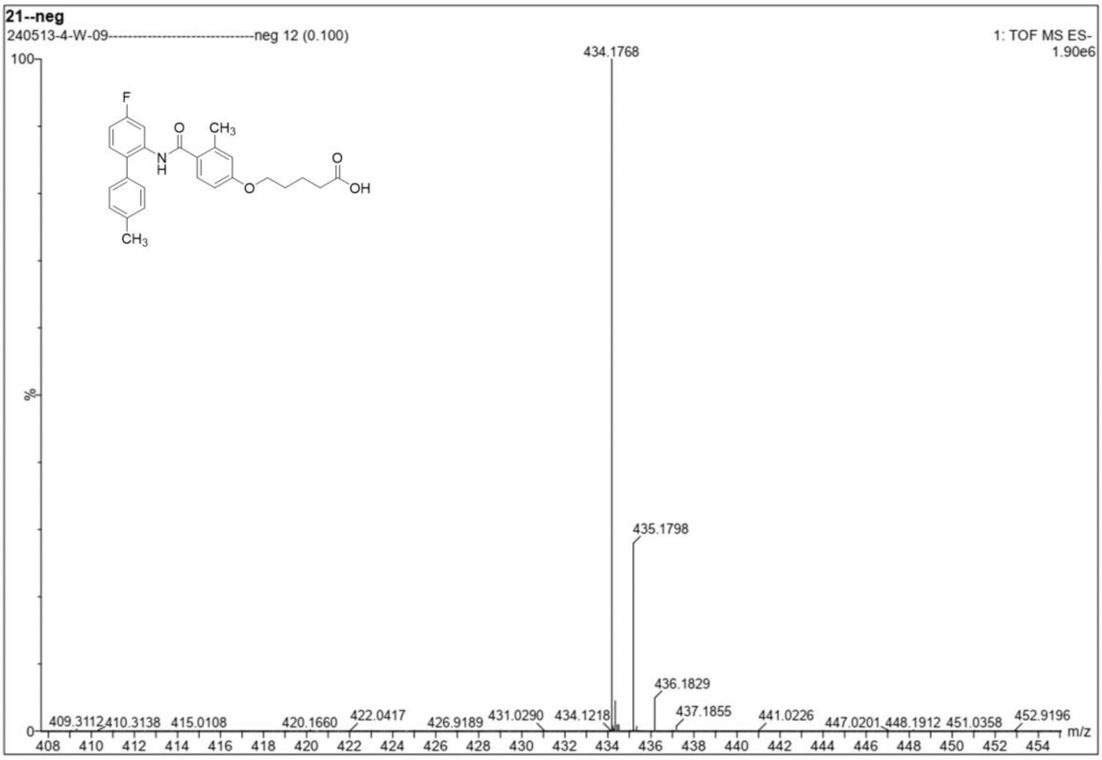

Figure S37. HRMS spectra of compound **9f**

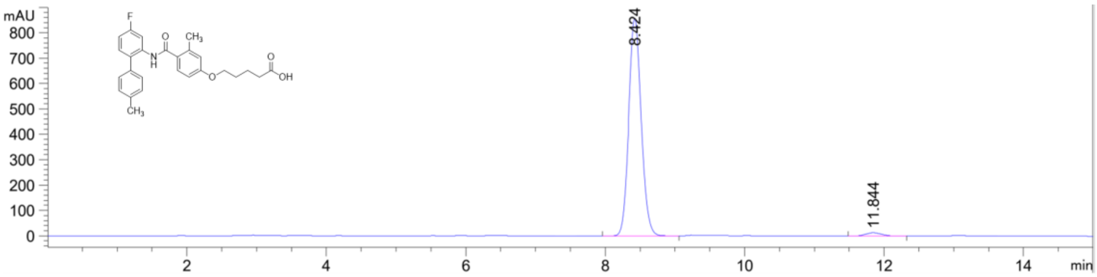

Peak	Retention time	Type	Peak width	Peak area	Peak height	Peak area ratio
#	[min]		[min]	[mAU*s]	[mAU]	%
1	8.424	BB	0.1908	1.06449e4	857.86432	98.1284
2	11.844	BB	0.2470	203.02534	12.80648	1.8716

Figure S38. HPLC spectra of compound **9f**

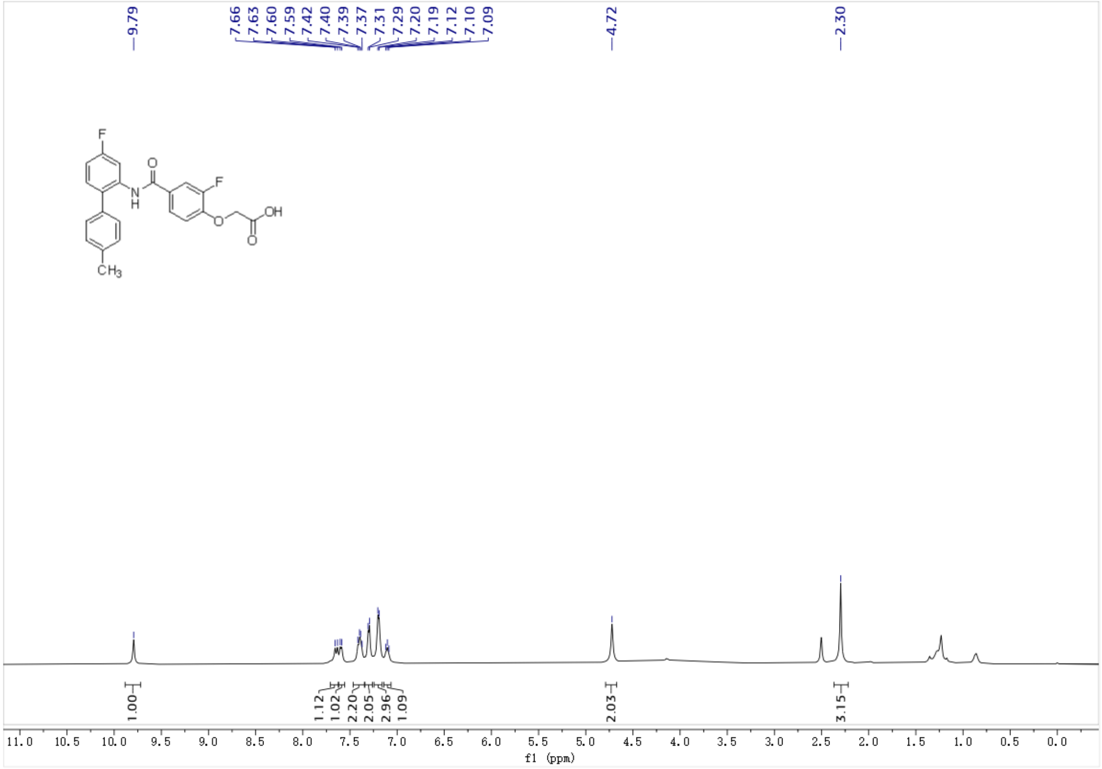

Figure S39. ^{1}H NMR spectrum for **10f** (DMSO-*d6*, 500 MHz)

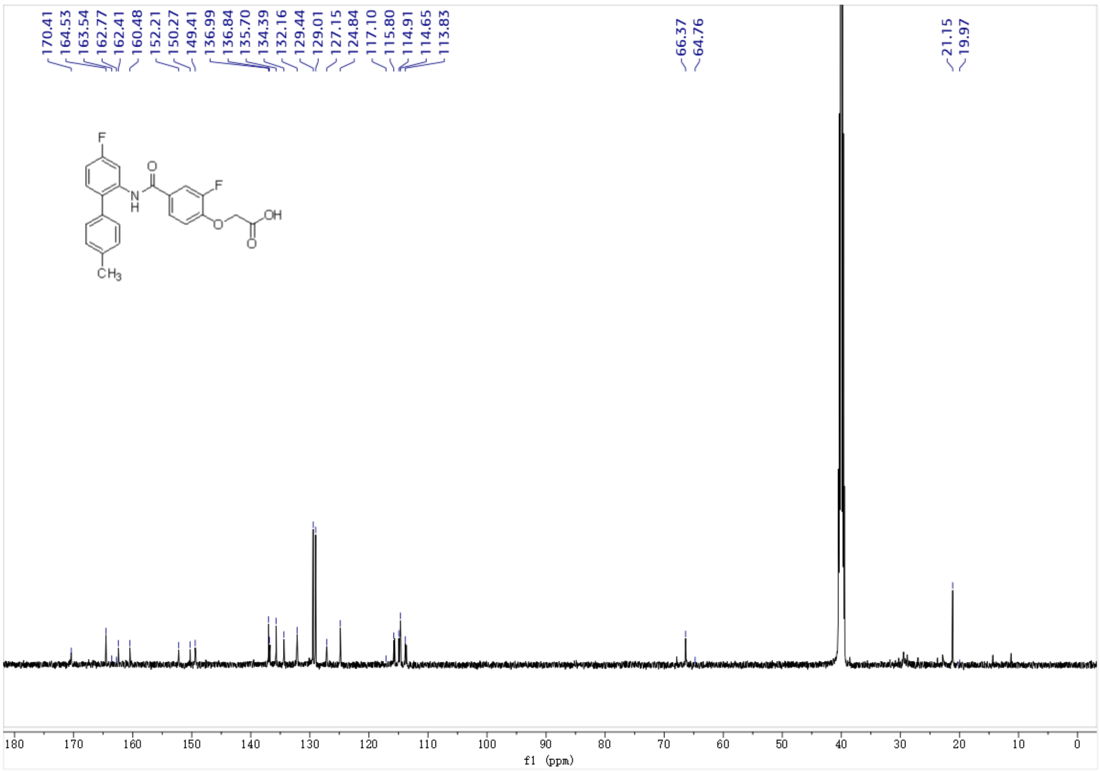

Figure S40. ^{13}C NMR spectrum for **10f** (DMSO-*d6*, 125 MHz)

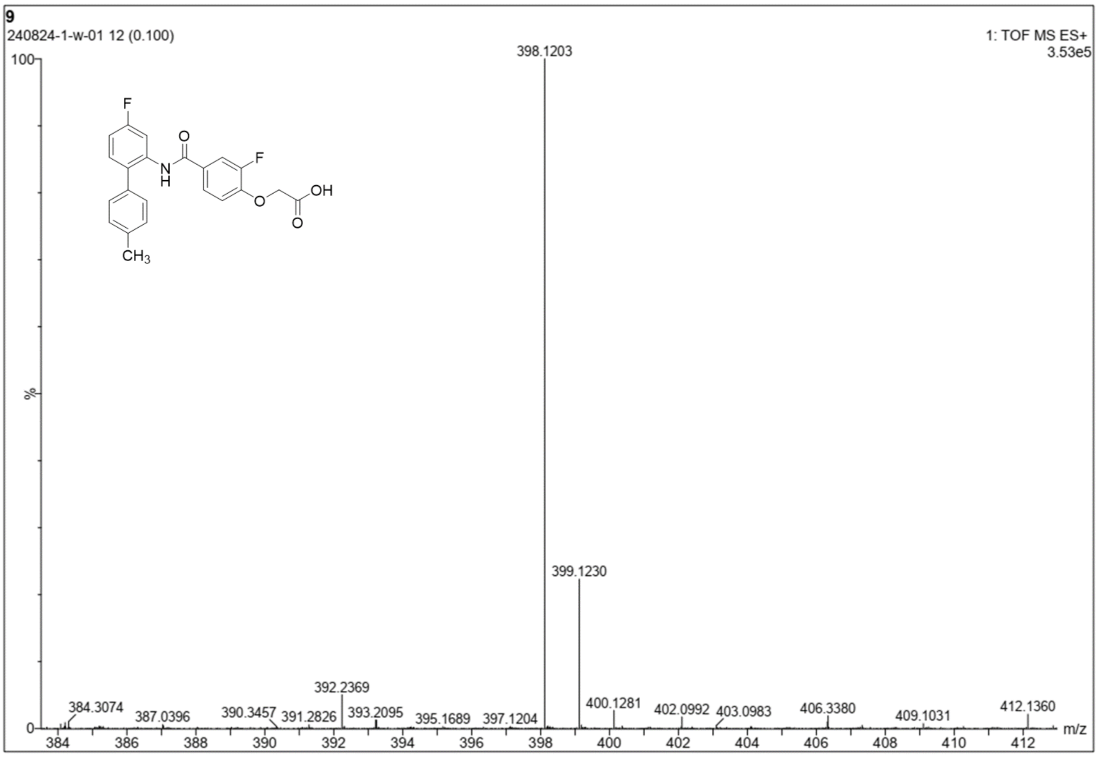

Figure S41. HRMS spectra of compound **10f**

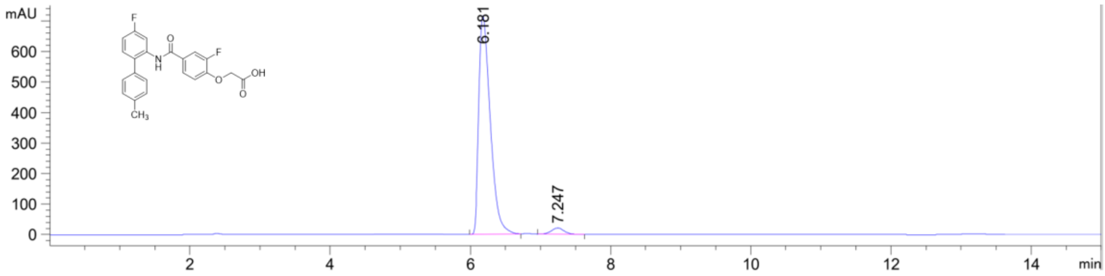

Peak #	Retention time [min]	Type	Peak width [min]	Peak area [mAU*s]	Peak height [mAU]	Peak area ratio %
1	6.181	BB	0.1744	8116.02734	715.12872	96.8304
2	7.247	BB	0.1977	265.66788	20.70150	3.1696

Figure S42. HPLC spectra of compound **10f**

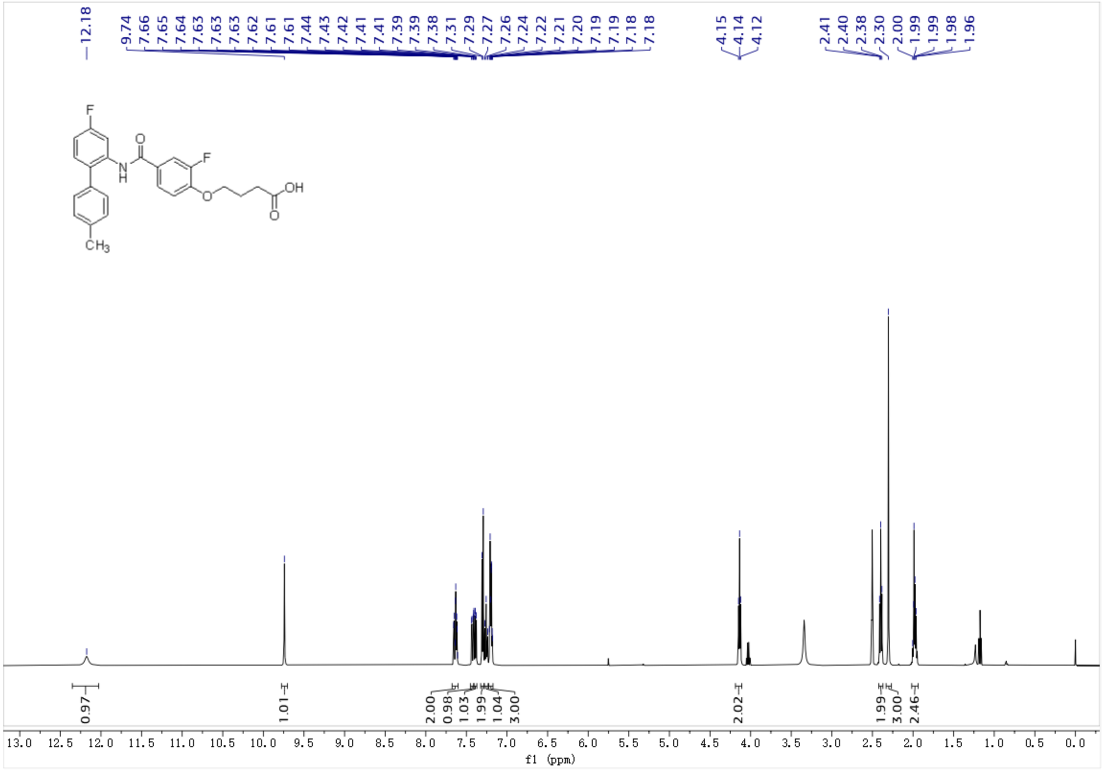

Figure S43. ^{1}H NMR spectrum for **11f** (DMSO-*d6*, 500 MHz)

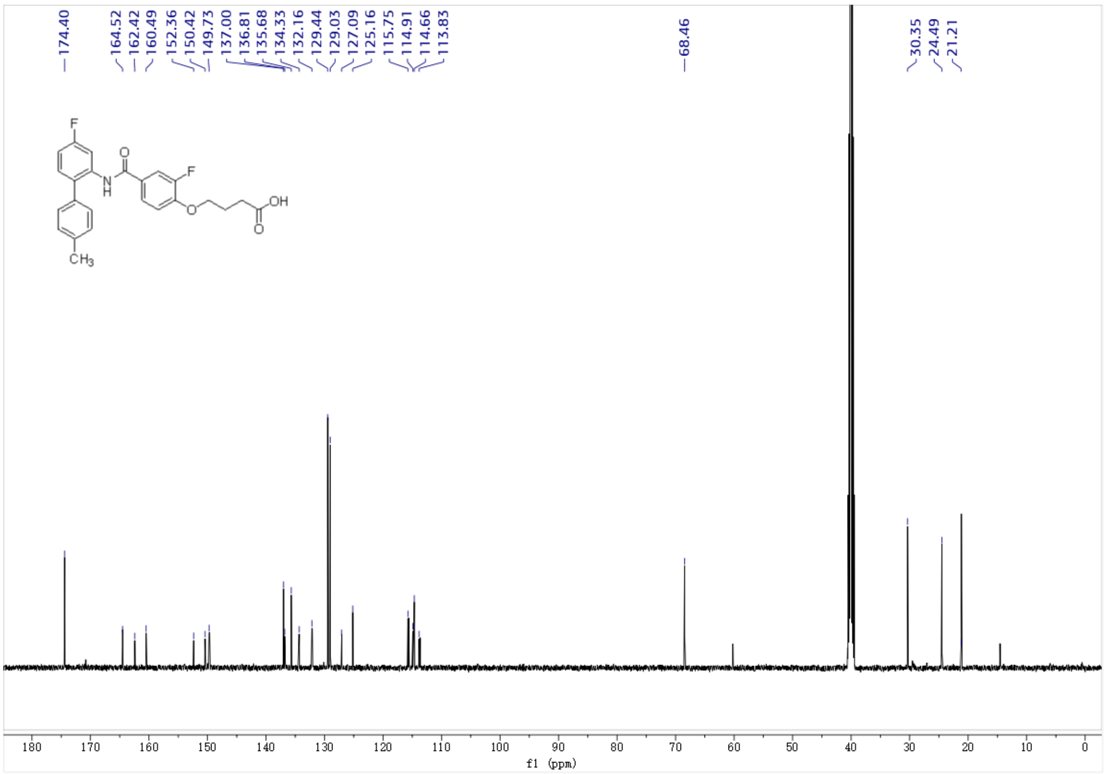

Figure S44. ^{13}C NMR spectrum for **11f** (DMSO-*d6*, 125 MHz)

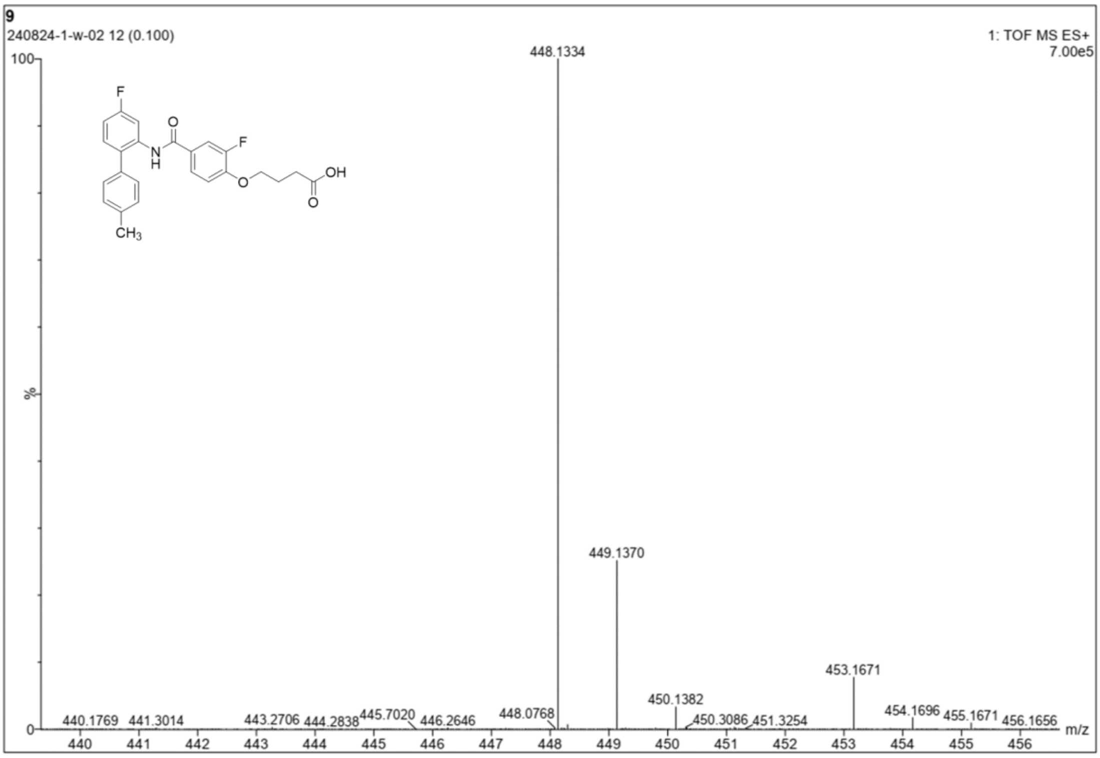

Figure S45. HRMS spectra of compound **11f**

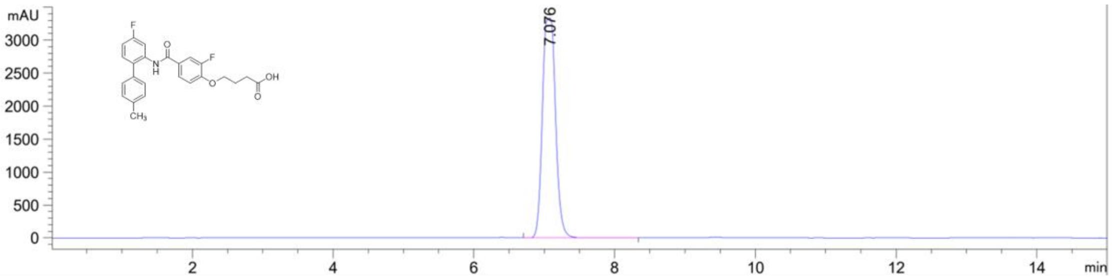

Peak #	Retention time [min]	Type	Peak width [min]	Peak area [mAU*s]	Peak height [mAU]	Peak area ratio %
1	7.076	BB	0.2075	4.39323e4	3338.45630	100.0000

Figure S46. HPLC spectra of compound **11f**

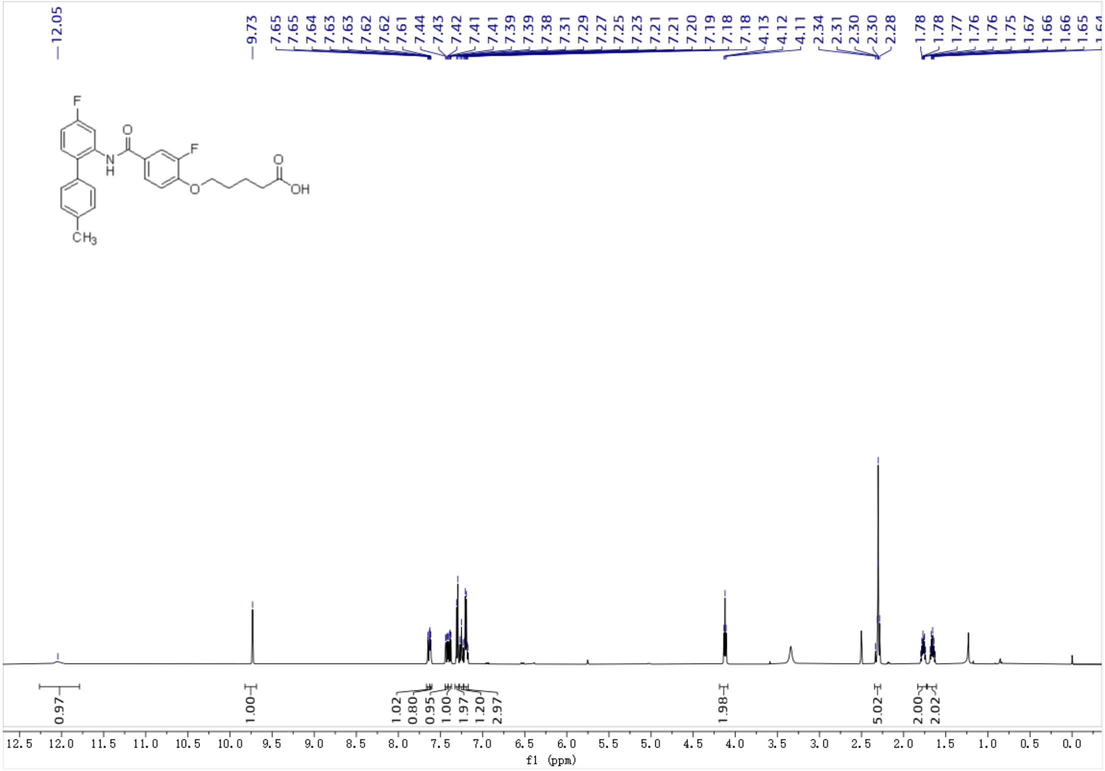

Figure S47. ^{1}H NMR spectrum for **12f** (DMSO-*d6*, 500 MHz)

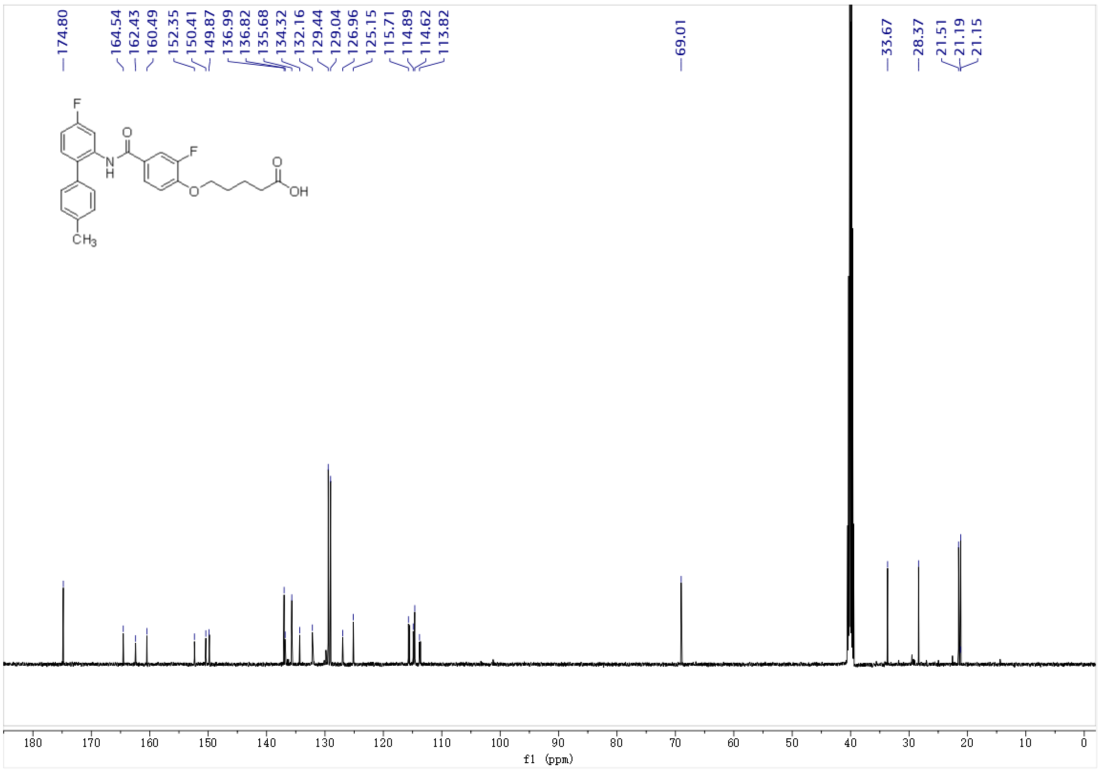

Figure S48. ^{13}C NMR spectrum for **12f** (DMSO-*d6*, 125 MHz)

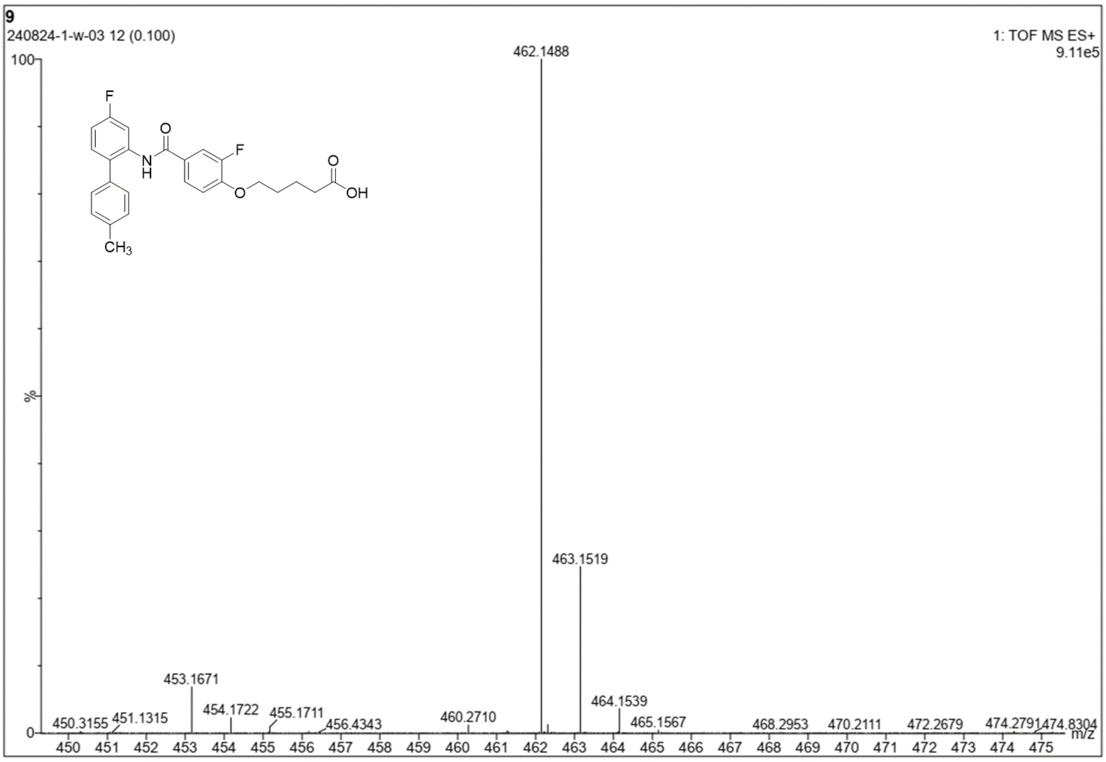

Figure S49. HRMS spectra of compound **12f**

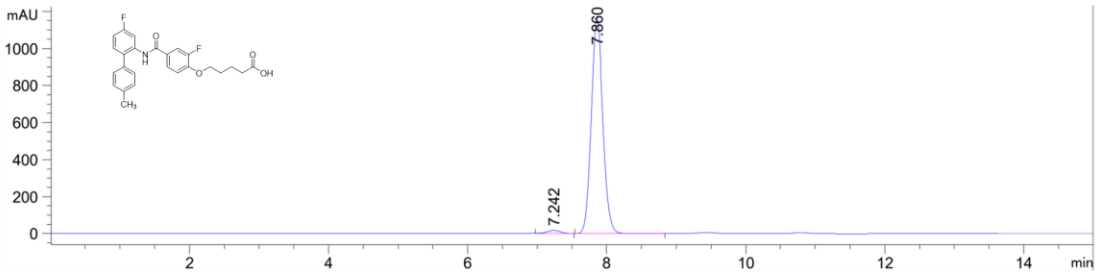

Peak #	Retention time [min]	Type	Peak width [min]	Peak area [mAU*s]	Peak height [mAU]	Peak area ratio %
1	7.242	BB	0.1993	215.23080	16.59283	1.5571
2	7.860	BB	0.1800	1.36072e4	1167.25317	98.4429

Figure S50. HPLC spectra of compound **12f**
